# Efficacy of electro-acupuncture in postpartum with diastasis recti abdominis: A randomized controlled clinical trial

**DOI:** 10.3389/fpubh.2022.1003361

**Published:** 2022-11-15

**Authors:** Yan Liu, Ying Zhu, Liyuan Jiang, Chao Lu, Lijuan Xiao, Ting Wang, Jiayu Chen, Li Sun, Lujun Deng, Meiyu Gu, Tingting Zheng, Min Feng, Yingying Shi

**Affiliations:** ^1^The Second Clinical Medical College, Zhejiang Chinese Medical University, Hangzhou, China; ^2^Zhejiang Chinese Medical University, Hangzhou, China; ^3^Department of Acupuncture and Rehabilitation, Hangzhou Hospital of Traditional Chinese Medicine, Hangzhou, China; ^4^Chun'an County Hospital of Traditional Chinese Medicine, Chun'an, China; ^5^Department of Rehabilitation Medicine, The Sixth Affiliated Hospital of Sun Yat-sen University, Guangzhou, China; ^6^Department of Maternal Health Care, Maternity and Child Health Care Centers of Hechi, Hechi, China; ^7^Dingqiao Hospital of Hangzhou Hospital of Traditional Chinese Medicine, Hangzhou, China

**Keywords:** acupuncture, diastasis recti abdominis, postpartum, intra-abdominal stimulation, randomized controlled trial

## Abstract

**Background:**

Electro-acupuncture (EA) has promising effects on diastasis rectus abdominis (DRA), defined as a separation of the two muscle bellies of rectus abdominis. To study, there is scant knowledge or scarce high-quality evidence.

**Objective:**

We aimed to evaluate the long-term efficacy and safety of EA in treating DRA during postpartum. It was assumed that the improvement of DRA was more obvious in the EA group than in the control group.

**Design:**

Randomized, controlled, blinded trial (Clinical Trial Registration: ChiCTR2100041891).

**Setting:**

Hangzhou Hospital of Traditional Chinese Medicine in China.

**Participants:**

Females aged 20–45 years without a past medical history of pathological rectus abdominal dissection were recruited from DRA inclusion criteria from 42 days to 1 year postpartum.

**Intervention:**

110 participants were randomly assigned in a 1:1 ratio to a control group with no EA intervention (*n* = 55), and EA group (*n* = 55). The EA group received ten sessions of EA combined with physical exercise or only physical exercise for 2 weeks with a 26-week follow-up.

**Measurements:**

Outcomes were assessed at baseline, week 2, and week 26. The primary outcome was the change of the inter recti distance (IRD) and electromyographic evaluation of the pelvic floor. Secondary outcomes included elasticity of linea alba (LA), paraumbilical subcutaneous adipose tissue (SAT) measurement, body mass index (BMI), percentage body fat (F%), dyspepsia symptoms, menstrual symptoms, quality of life (QoL), pain performance of patients with lower back pain, postnatal depression symptoms (PDS), postpartum self-image, and DRA-related symptom assessment including urine leakage, frequency, and urgency, constipation, sexual dysfunction, and chronic pelvic pain.

**Results:**

A total of 110 maternal (55 in each group) were recruited. The mean difference in IRD from baseline to week 2 and week 26 in all states of the two groups were reduced compared with those before treatment, with statistical significance (*P* < 0.05). The mean of IRD at the horizontal line of the umbilicus in the end-expiratory state was smaller in the EA group than in the control group, but the difference was not statistically significant (*P* > 0.05) at week 2. The mean of IRD at the horizontal line of the umbilicus in head-up and flexed knee state was smaller in the EA group than in the control group, and the difference was statistically significant (*P* < 0.05) at week 26. Five (9.1%) and thirteen (23.64%) adverse events were reported in EA and control groups, respectively. No serious adverse events were reported.

**Limitation:**

The frequency intensity of EA parameters was selected between 4 and 6 because of individual tolerance differences.

**Conclusion:**

EA is an effective approach to improve IRD, electromyographic evaluation of the pelvic floor, BMI, the elasticity of LA, paraumbilical SAT, and symptoms of DRA, with durable effects at 26 weeks.

**Primary funding source:**

The Construction Fund of Medical Key Disciplines of Hangzhou (Project Number: OO20200097), Hangzhou Medical and Health Science and Technology Project No. A20200483, and Zhejiang Traditional Chinese Medicine Science and Technology Plan Project (Project Number: 2021ZQ065).

**Clinical trial registration:**

http://www.chictr.org.cn/index.aspx, identifier: ChiCTR2100041891.

## Introduction

Diastasis recti abdominis (DRA) is defined as a separation of the rectus abdominal muscles disintegrating to the sides, accompanied by the extension of the linea alba (LA) tissue and bulging of the abdominal wall (1, 2). Diastasis recti abdominis is diagnosed when the inter-rectus distance is > 2 cm (3, 4). It affects 30–70% of women during pregnancy (5), and 35–70% of pregnant women do not recover after giving birth without treatment or exercise (6). In addition, 39–45% of women continue to have DRA at 26 weeks postpartum, and the incidence of DRA at 1 year postpartum is 23–32% (1). The negative effects of DRA manifest in physical function, abdominal trunk function, and impairment of quality of life (QoL) in postpartum women. Women with DRA primarily receive the application of support band and abdominal band during pregnancy and postpartum (6), electrical stimulation, surgical repair (7), and physical exercise (8). There is a lack of a unified and effective treatment plan. There are few studies on the efficacy and safety of current treatments (7, 9); careful follow-up for adverse events must be considered with long-term use. As a worldwide alternative therapy, acupuncture has received wide attention in preventing and treating issues related to pregnancy and childbirth.

Acupuncture therapy is rooted in a complex practice ritual, especially the acupuncture needle procedure, particularly when coupled with EA stimulation. Electro-acupuncture applies electrical stimulation to acupuncture needles (10), which generates improved tissue excitability (11) and adjusts the mechanical balance of the postpartum abdominal muscle group. However, the long-term efficacy of EA is still unclear, and there is a lack of solid objective evidence. To date, there are no RCT studies on the impact of EA on DRA or evaluating the standardized EA application for DRA. This study comprehensively evaluates the effectiveness and safety of EA in the treatment of postpartum DRA. It provides a reference for the clinical treatment of postpartum DRA.

## Methods

### Design overview

This was a single-center, randomized, and controlled clinical trial, following the Consolidated Standards of Reporting Trials (CONSORT) statement (12), the Standardized Protocol Items: Recommendations for Interventional Trials (SPIRIT) guidelines (13), and the Revised Standards for Reporting Interventions in Clinical Trials of Acupuncture (STRICTA) (14). It involved females aged 20–45 years without a past medical history of pathological rectus abdominal dissection, who were recruited from DRA inclusion criteria in 42 days to 1 year postpartum.

The trial was carried out in accordance with the Declaration of Helsinki (15). The Ethics Committee of Hangzhou Hospital of Traditional Chinese Medicine reviewed this study's protocol and gave its approval and consent (approval code 2020KY082, Supplementary material 1), which agreed with the Declaration of Helsinki (Version Fortaleza 2012). Clinical Trial Registration: Chinese Clinical Trial Registry, ChiCTR2100041891. All data generated or analyzed in this study will be fully available without restriction through the Clinical Trial Management Public Platform (www.medresman.org.cn, Supplementary material 2). All study patients provided informed consent.

### Sample size

According to previous similar reports (16), the mean value of inter recti distance (IRD) in the control group was 2.09 after treatment. The mean value of IRD in the EA group was expected to be 1.43 after treatment in this study. Two groups were set up in this study. The test level was α = 0.05 with a test efficiency of 1 – β = 0.90. A two-sided test was also conducted. PASS (Power Analysis and Sample Size) 15.0 software (17) estimated the sample size and effect size as 0.313269. Considering 2-sided *P-*values to be deemed statistically significant at *P* < 0.05 and a power of 90%, 50 patients would be required per group (NQuery Advisor, version 4.0; Statistical Solutions). Estimating that 10% of patients might be lost to follow-up, we planned to enroll 110 patients, with 55 in each group.

### Setting and patients

The study was conducted in the outpatient department of Hangzhou Hospital of Traditional Chinese Medicine. Volunteers were recruited *via* hospitals' WeChat (Version 8.0.27) public platform and hospital posters. Patients were recruited using the following inclusion criteria:

(1) Female aged 20–45 years;
(2) 42 days to 1 year postpartum;
(3) The use of ultrasound to evaluate DRA (18) in (a) the midpoint of the umbilicus and xiphoid process, (b) the horizontal line of the umbilicus, and (c) the midpoint of the umbilical and pubic symphysis line. If at any point of the three measurements, IRD is ≥2 cm (3) at the resting state;
(4) No cognitive barriers, and able to understand and communicate correctly;
(5) Those who sign the informed consent, cooperate with the treatment, and commit to completing all therapy as planned.

Note: Patients who met the above five criteria were included in this study.

The study also had the following exclusion criteria:

(1) Patient is suspected or diagnosed with severe spinal lesions (such as spinal fractures, metastases, inflammatory or infectious diseases, or cauda equina syndrome/widespread neurological disease) and neurological injury.
(2) Patient has motor contraindications or severe infectious diseases such as fractures, severe heart disease, hypertension, and cancer.

Patients with any of the above were to be excluded.

### Randomization and masking

Eligible patients were randomly assigned in a 1:1 ratio to EA or control group *via* a random-number table (Supplementary material 3) to balance known and unknown confounding factors and thus improve comparability between the two groups. The third-party operator (Lijuan Xiao) put the grouping list into a sequentially numbered, opaque, sealed envelope and delivered it to the operator (Li Sun) to complete the subject intervention assignment. The study leader (Liyuan Jiang) generated allocation numbers, Ying Zhu recruited subjects, and Li Sun assigned interventions. Patient recruiters, outcome assessors, and statisticians did not touch these envelopes until data processing was complete. Participants and the acupuncture provider were not blind to the groups because of the specificity of the EA treatment (19). Outcome assessors, physical therapists (PT), and statisticians were blinded to treatment assignments. Guesstimates of EA group assignment were completed by outcome assessors, PT, and statisticians at the end of the study follow-up. Statistical blinding assessments were performed using the Bang's index and James index (20).

### Interventions

The intervention protocol was based on the previous literature and clinical experience of DRA (21). The treatment was administered by a certified acupuncturist (Yingying Shi) who had 23 years of clinical experience in EA. The selection of acupoints was based on Chinese literature and clinical experience. The acupuncture locations are described in The National Standards for Acupoint Location (22).

**Figure 1:**
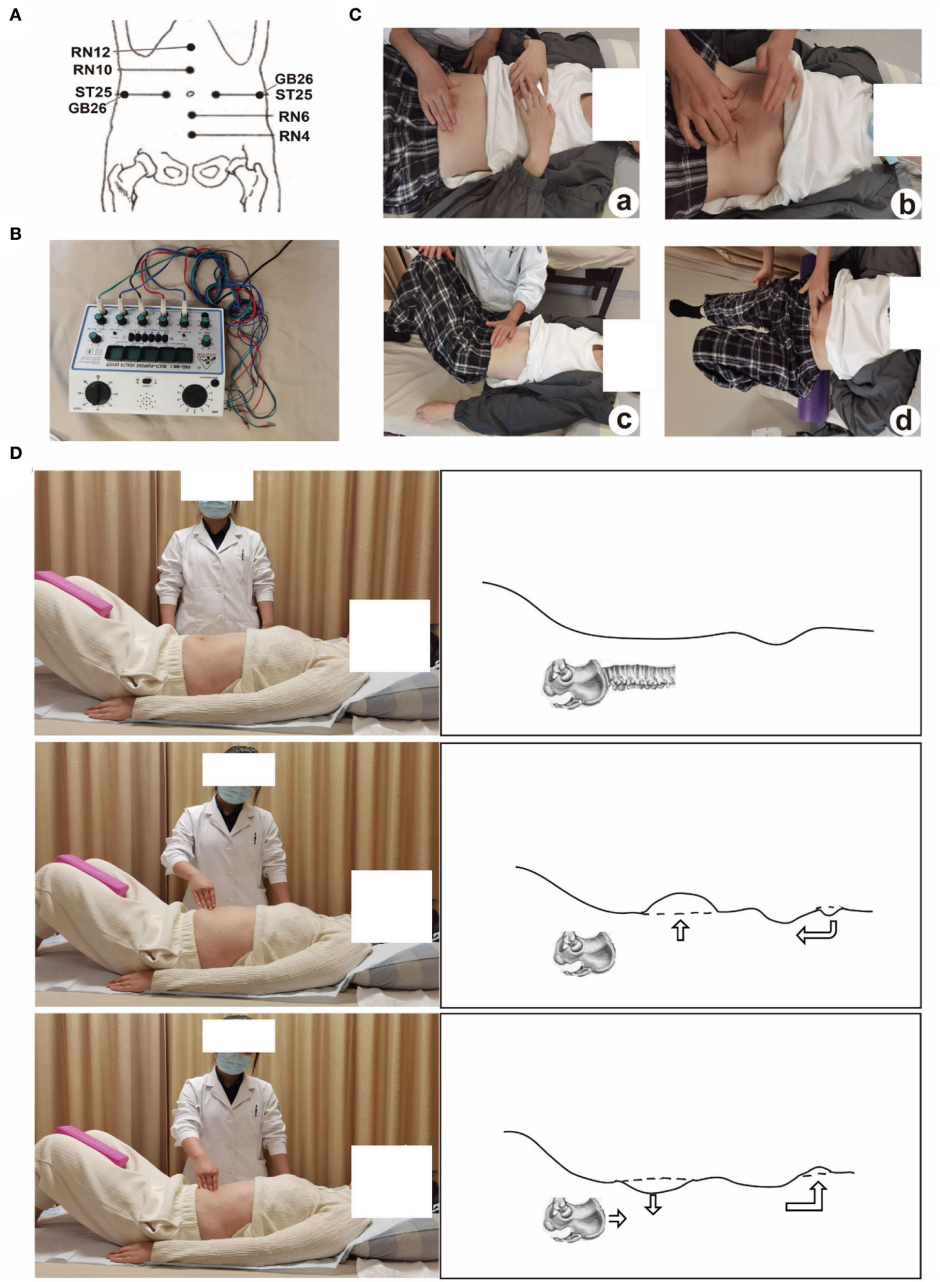
**(A)** the location of the acupoint; **(B)** the EA instrument Great Wall KWD-808I; **(C)** the graphic representation of physical exercise; **(D)** the fascial abdominal breathing at rest (from the authors' own archives, reprinted with the patient's permission).

For the EA group (electro-acupuncture + physical exercise), the patient was placed in the supine position, exposing the abdomen and acupoints Zhongwan (RN12), Xiawan (RN10), bilateral Tianshu (ST25), bilateral Dai Mai (GB26), Qi Hai (RN6), and Guanyuan (RN4) were selected (Figure 1A).

The skin at the acupoints was routinely disinfected, and disposable sterile acupuncture needles were used for vertical acupuncture of 25–40 mm. The acupoints were Zhongwan (RN12), Xiawan (RN10), bilateral Tianshu (ST25), bilateral Dai Mai (GB26), Qi Hai (RN6), and Guanyuan (RN4). The needles were manipulated until the patient felt a “de qi” sensation (23), and were connected to EA (the instrument was Great Wall KWD-808I (Figure 1B) continuous wave (CW) tuning knob of pulse rate “2”. The intensity was adjusted to 4–6 mA, which was appropriate if the abdominal muscles contract without feeling pain. The treatment was for 30 min once/day, five times a week for 2 weeks. Physical exercise was the same as the control group.

The control group received the following (only physical exercise, Figure 1C): (a) Fascial abdominal breathing (Figure 1D): The patient was kept at the supine position, lower limb hip, and knee flexion, with foam bricks clamped between the legs. The abdomen was humped when inhaling and was forced to the navel when exhaling. Abdominal muscles and pelvic floor muscles was forced to contract at the same time. This was to be repeated ten times per set and a total of three sets for this exercise. (b) Supine head training: The patient was asked to assume a supine position, lower limb hip, and knee flexion, with foam brick between the legs, and directed to do abdominal breathing increasing abdominal muscle contraction force during exhalation. The head was then held up, and the parts below the lower edge of the scapula cannot leave the bed surface. This was to be repeated ten times per set and a total of three sets for this exercise. (c) Left and right-side leg rotation: The patient was asked to adopt a buckling posture, supine, and legs down to the right. The patient was then asked to inhale with the abdominal bulge, exhale with abdomen muscle contraction, and move both legs in a buckled posture to the left (engage the core abdominal muscles and not engage excessive leg muscles.). The therapist placed one hand on the right side of the external oblique muscle of the patient during muscle contraction, and with the other hand, the therapist applied counter resistance at the side of the knees according to the strength of the patient's exertion. The patient was to repeat this movement alternating on both sides and do it ten times each. (d) Supine cycling: In the supine position, with foam placed at the lumbosacral axis, and arms on both sides of the body, the patient was asked to lift the legs off the bed surface and perform a cycling action. The patient had to complete the cycling action ten times for one set and repeat the set three times. Each exercise was designed for about 5 min, and a total of 20 min, once/day, five times a week for 2 weeks. Patients in both groups started their treatment on the day of randomization and received ten sessions for two consecutive weeks: 5 sessions every week (ideally five consecutive days) until ten sessions. All patients were followed up for 26 weeks.

The same acupuncturist (Yingying Shi) delivered the treatment with standardized operating procedures (Figures 1A,B). Patients were encouraged to refrain from using other therapies for the management of DRA throughout the trial. If other therapies were used, details were documented on a concomitant therapy form. Any adverse event (AE), or side effects (SE) (e.g., bleeding, post stitch, needle blocking) were to be documented in detail on the form and reported to the project leader (Liyuan Jiang). Serious adverse events were to be immediately reported to the institutional review board at the clinical sites within 26 h. Subjects with adverse events were to be treated in the hospital where the project was being implemented, and the project team was to bear the treatment and examination costs.

### Assessments and outcomes

The primary outcome was the amelioration of the inter recti distance (IRD) determined by ultrasound at weeks 2 and 26. The response was assessed immediately after the 2-week treatment (week 2) and 24 weeks after treatment (week 26). The between-group difference had to be statistically significant at both time points for us to conclude the efficacy for at least 26 weeks.

IRD is the distance between the rectus abdominal muscles (18). An ultrasound scanner (LOGIQ E9) with a 6–15 MHz high-frequency probe with ML6-15 was used to collect images (MSK Gen mode). Patients were asked to take the supine position and fully expose the upper abdomen. Three measurement sites were selected (the midpoint of the umbilicus and xiphoid process, the horizontal line of the umbilicus, and the midpoint between umbilicus and pubic symphysis at resting state), and wide-field imaging was used when necessary. The mean value of three results from each was taken as the reference value.

IRD and electromyographic evaluation of the pelvic floor by Creative Medical Biofeedback System (AM1000B) were evaluated as the primary endpoint using an ultrasound (18).

The secondary outcomes included: (1) The elasticity of linea alba is assessed by strain elastography (24). The elastic mode is selected at two sites (the horizontal line of the umbilicus, and the midpoint of the umbilicus and xiphoid process). The elastic zone of interest includes the LA and surrounding tissues, and the zone of interest is adjusted to more than twice the area of the LA and as far as possible the mass scale color is kept fluctuating smoothly within the range of 1/3 to 2/3. Mass scale yellow or green is preferred. The smoothness lasts at least 5s. (2) Body mass index (BMI); (3) Paraumbilical subcutaneous adipose tissue (SAT) measurement (25); (4) Percentage body fat (F%) (26).

Other outcomes: (1) Dyspepsia symptoms were evaluated using the Leeds dyspepsia questionnaire (LDQ). LDQ has six grades based on the severity and frequency of the symptoms. The higher the score is, the more serious the symptoms are. LDQ has qualified validity, reliability, reactivity, and internal unity. Therefore, this study chose LDQ as the evaluation index of dyspepsia symptoms to evaluate the difference in efficacy of electro-acupuncture and the control group in treating DRA from the improvement of dyspepsia symptoms. (2) Menstrual symptoms were measured by the Menstrual Distress Questionnaire (25). (3) Quality of life (QoL) was assessed by the Short Form 36 (SF-36^®^) questionnaire (27, 28) where eight dimensions of health-related quality of life are assessed: physical functioning (PF), role-physical (RP), bodily pain (BP), general health (GH), vitality (VT), social functioning (SF), role-emotional (RE) and mental health (MH). In addition, the reported health transition (HT) is included. (4) Overall improvement as measured by the short-form McGill pain questionnaire (SF-MPQ) or symptom scale based on the Clinical Study Guideline for New Developed Chinese Medicine (29–31). The questionnaire can be used to assess the pain performance of patients with lower back pain, including the Pain Rating Index (PRI) calculated from the SF-MPQ scale where the PRI is the sum of sensory item scores and emotional item scores; the Visual Analog Scale (VAS) (27, 32); and the Present pain intensity (PPI). (5) Postnatal depression symptoms (PDS) were assessed with Edinburgh postnatal depression scale (EPDS). (6) Postpartum self-image was assessed using the Modified Body Self-Image Scale (MBIS). (7) DRA-related symptom assessment of urine leakage, frequency, and urgency; constipation; sexual dysfunction; and chronic pelvic pain. (8) The main idea of the Hernia-Related Quality of Life Survey (HerQLes) (33) questionnaire was adapted to ask subjects how they felt about the separation of the rectus abdominis muscle and how it affected their lives.

For the evaluation of compliance and adverse events, the patients were instructed to perform physical exercise every day for 26 weeks. Their compliance (number of physical exercises per day, duration of physical exercise per day, movements per day, reasons for not being able to adhere to them) and other conditions (whether they had received other treatment for rectus abdominal separation in the past 26 weeks, whether they had received related treatment for other diseases in the past 26 weeks, whether they had weight-bearing exercises and the frequency of weight-bearing in the past 26 weeks) were statistically evaluated at the end of the follow-up period (Table 1).

**Table 1:** The analysis of compliance.

**Questions**	**Grade**	**EA group**	**Control group**	***P-*value**
Number of exercises per day	0 time	19 (35.20%)	19 (36.50%)	0.479
	< 1 time on average	27 (50.00%)	30 (57.70%)	
	1 time	7 (13.99%)	3 (5.80%)	
	2 times	1 (1.90%)	0 (0.00%)	
	3 times	1 (0.50%)	0 (0.00%)	
	>3 times	0 (0.00%)	0 (0.00%)	
Daily exercise movements (multiple choice)	No	20 (37.00%)	19 (36.50%)	0.958
	Fascial abdominal breathing	32 (59.30%)	32 (61.50%)	0.811
	Supine head training	6 (11.10%)	5 (9.6%)	0.802
	Left and right-side leg rotation	4 (7.40%)	2 (3.80%)	0.430
	Supine cycling	3 (5.60%)	2 (3.80%)	0.680
Daily exercise time	0	19 (35.20%)	18 (34.60%)	0.647
	< 5 min	14 (25.90%)	17(32.70%)	
	5-10 min	12 (22.20%)	11 (21.20%)	
	10-20 min	6 (11.10%)	5 (9.60%)	
	> 20min	3 (5.60%)	1 (1.90%)	
Reasons for not being able to exercise consistently (multiple choice)	Forget	27 (50.00%)	30 (57.70%)	0.429
	No time	31 (57.40%)	39 (75.00%)	0.057
	Unwillingness	7 (13.00%)	12 (23.10%)	0.177
	Not necessary	1 (1.90%)	0 (0.00%)	0.326
	Not mastering the method	0 (0.00%)	0 (0.00%)	1.000
Any other treatment for separation of the rectus abdominis muscle in the last 24 weeks	No	51 (94.40%)	47 (90.40%)	0.431
	Yes	3 (5.60%)	5 (9.60%)	
Any related treatment for other illnesses in the last 6 months	No	51 (94.40%)	43 (82.70%)	0.057
	Yes	3 (5.60%)	9 (17.30%)	
Any weight-bearing activities (carrying children/heavy objects) in the last six months	No	4 (7.40%)	10 (19.20%)	0.113
	Yes	50 (92.60%)	42 (80.80%)	
Weight frequency, if any	≥20 times/week	39 (78.00%)	38 (90.50%)	0.109
	< 20 times/week	11 (22.08%)	4 (9.50%)	

### Statistical analysis

Data were analyzed using Python 3.8 software. Categorical variables were presented by frequency (percentage) and analyzed with the chi-squared test or Fisher's exact test. If they met normal distribution, continuous variables were presented as mean ± standard deviation (M ± SD). Otherwise, they were presented as medians ± interquartile range (M ± IQR). The demographic characteristics were compared between the groups by independent *t*-tests at baseline. To evaluate the safety of acupuncture, we used a Fisher exact test to report the relative risk of an adverse effect. Analysis of the correlation between the elasticity of linea alba and IRD was undertaken using Spearman's correlation analysis. All tests were two-sided, and a *P*-value of < 0.05 was considered statistically significant.

## Results

### Patients

**Figure 2:**
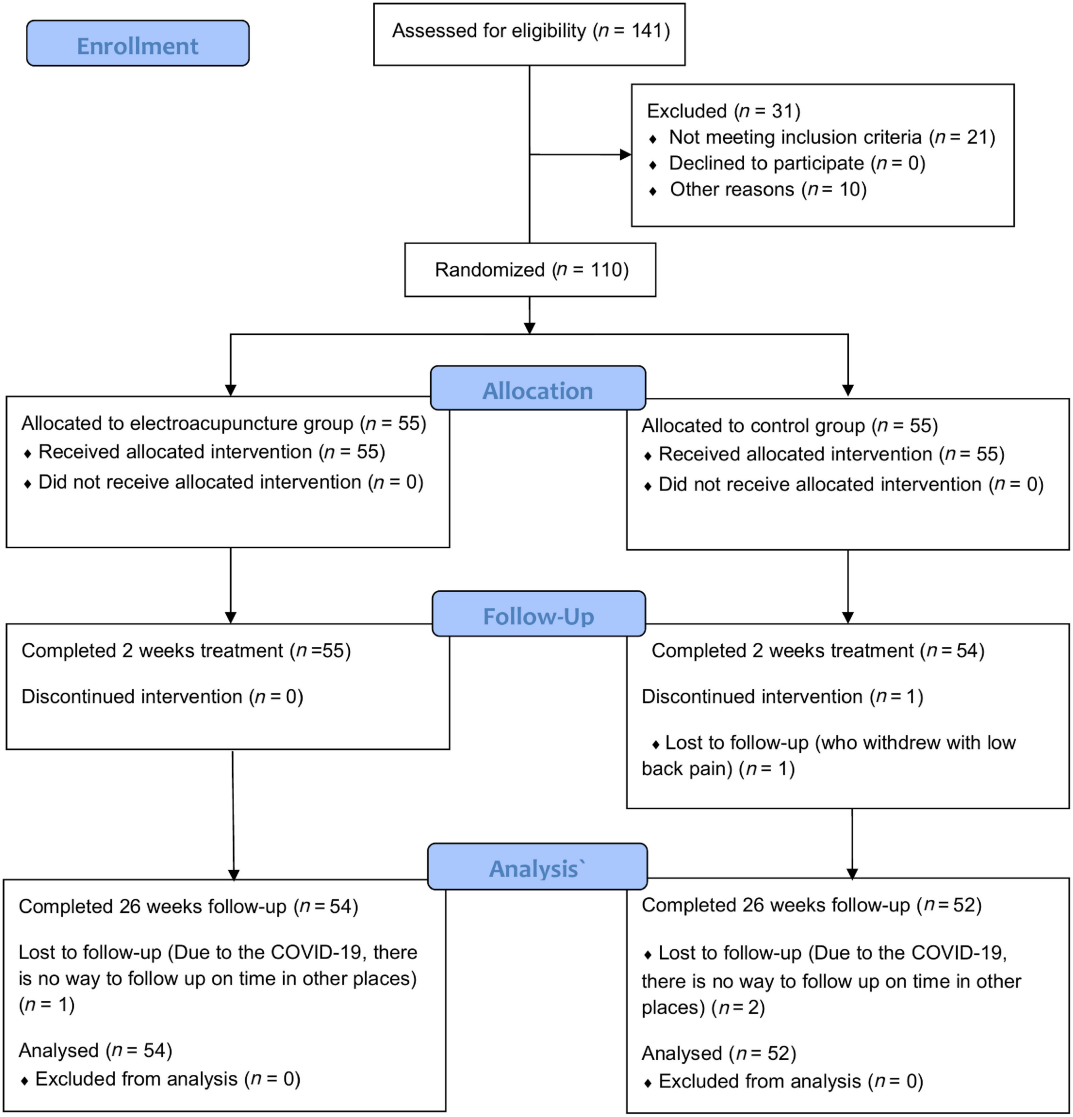
Study flow diagram of participants through the study period.

The study's flow chart is shown in Figure 2. Between 18 January 2021 and 24 January 2022. A total of 31 patients were not enrolled, of whom 21 (67.7%) met exclusion criteria and 10 (32.3%) were eligible but not enrolled for other reasons (Figure 2). A total of 110 randomized patients enrolled in the study of which 55 were randomized to the EA group and 55 to the control group. Only one patient (1 [who withdrew with low back pain] in the control group) did not receive the study's consecutive treatment. The follow-up to 26 weeks was incomplete for 3 patients (due to COVID-19, there was no way to follow up on time in other places). Thus, data for 106 patients (54 in the EA group and 52 in the control group) were used in the final analysis (Figure 2). Attendance in the study was similar between groups.

Baseline characteristics are presented in Table 2. There were no differences between the two groups regarding patient characteristics, IRD, LDQ, and menstrual symptoms as measured by the Menstrual Distress Questionnaire, QoL, EPDS, and so on.

**Table 2:** Baseline characteristics of the study population^*^.

**Characteristic**	**All**	**EA**	**Control**	***P*-value**
	**(*n =* 110)**	**(*n =* 55)^†^**	**(*n =* 55)^†^**	
**Age, y**				0.054
N (Nmiss)	110 (0)	55 (0)	55 (0)	
Mean±SD	32.60 ± 3.93	32.56 ± 4.27	32.77 ± 3.58	
Min–Max	24–42	24–42	23.61–40.43	
Median (IQR)	32.0 (5.0)	32.10 (6.78)	33.44 (4.90)	
**Height, m**				0.443
N (Nmiss)	110 (0)	55 (0)	55 (0)	
Mean±SD	160.35 ± 4.88	160.00 ± 5.33	160.69 ± 4.41	
Min–Max	150–171	150–171	150–170	
Median (IQR)	160 (7.3)	160 (8)	160 (6)	
**Mean weight before this pregnancy, kg**				0.268
N (Nmiss)	110 (0)	55 (0)	55 (0)	
Mean±SD	53.92 ± 6.97	52.98 ± 7.20	54.85 ± 6.67	
Min–Max	42–75	42–70	42–75	
Median (IQR)	53 (11)	52 (9)	55 (10)	
**Weight before this prenatal, kg**				0.823
N (Nmiss)	110 (0)	55 (0)	55 (0)	
Mean±SD	68.51 ± 8.07	67.71 ± 7.96	69.31 ± 8.17	
Min–Max	52–98	53–89	52–98	
Median (IQR)	68.0 (8.9)	68 (9.2)	70 (8)	
**Weight after childbirth, kg**				0.729
N (Nmiss)	110 (0)	55 (0)	55 (0)	
Mean±SD	61.32 ± 7.95	59.99 ± 7.39	62.65 ± 8.32	
Min–Max	44–89	44–80	46–89	
Median (IQR)	60.0 (9.25)	60.0 (10.5)	62.0 (10.0)	
**Mean BMI before this pregnancy, kg/m** ^**2**^				0.598
N (Nmiss)	110 (0)	55 (0)	55 (0)	
Mean±SD	20.99 ± 2.41	20.67 ± 2.42	21.48 ± 2.86	
Min–Max	16.41–27.34	17.01–27.34	16.41–31.22	
Median (IQR)	20.50 (3.06)	20.31 (2.77)	21.64 (4.02)	
**BMI before this prenatal, kg/m** ^**2**^				0.921
N (Nmiss)	110 (0)	55 (0)	55 (0)	
Mean±SD	26.62 ± 2.68	26.42 ± 2.53	27.18 ± 3.86	
Min–Max	20.96–35.56	20.96–33.20	20.31–40.79	
Median (IQR)	26.37 (2.92)	25.89 (2.52)	27.24 (4.86)	
**BMI after childbirth, kg/m** ^**2**^				0.453
N (Nmiss)	110 (0)	55 (0)	55 (0)	
Mean±SD	23.83 ± 2.74	23.40 ± 2.41	24.57 ± 3.83	
Min–Max	17.42–32.30	18.31–28.76	18.07–37.04	
Median (IQR)	23.44 (3.35)	23.03 (2.88)	24.38 (4.44)	
**Baby's birth weight, kg**				0.330
N (Nmiss)	110 (0)	55 (0)	55 (0)	
Mean±SD	3.42 ± 0.51	3.42 ± 0.47	3.42 ± 0.55	
Min**–**Max	2.07**–**6.0	2.07**–**4.56	2.5**–**6.0	
Median (IQR)	3.4 (0.7)	3.42 (0.71)	3.40 (0.64)	
**Delivery mode**, ***n*** **(%)**				0.529
Spontaneous vaginal delivery	59 (53.6)	28 (50.9)	31 (56.4)	
Cesarean section	51 (46.4)	27 (49.1)	24 (43.6)	
**Past medical history**, ***n*** **(%)**				0.782
Yes	15 (13.6)	7 (12.7)	8 (14.5)	
No	95 (86.4)	48 (87.3)	47 (85.5)	
**Medication history**, ***n*** **(%)**				0.697
Yes	7 (6.4)	3 (5.5)	4 (7.3)	
No	103 (93.6)	52 (94.5)	51 (92.7)	
**Allergic history**, ***n*** **(%)**				0.142
Yes	13 (11.8)	4 (7.3)	9 (16.4)	
No	97 (88.2)	51 (92.7)	46 (83.6)	
**Previous abdominal surgery**, ***n*** **(%)**				0.708
Yes	55 (50.0)	30 (54.5)	25 (45.5)	
No	55 (50.0)	25 (45.5)	30 (54.5)	
**Number of pregnancies**				0.868
N (Nmiss)	110 (0)	55 (0)	55 (0)	
Mean±SD	1.91 ± 1.09	1.93 ± 1.21	1.89 ± 0.96	
Min**–**Max	1**–**7	1**–**7	1**–**5	
Median (IQR)	2 (1)	1 (1)	2 (1)	
**Number of deliveries**				0.478
N (Nmiss)	110 (0)	55 (0)	55 (0)	
Mean±SD	1.47 ± 0.57	1.45 ± 0.57	1.49 ± 0.57	
Min**–**Max	1**–**3	1**–**3	1**–**3	
Median (IQR)	1 (1)	1 (1)	1 (1)	
**Multiple or twin pregnancies**, ***n*** **(%)**				0.978
Yes	2 (1.8)	1 (1.8)	1 (1.8)	
No	108 (98.2)	54 (98.2)	54 (98.2)	
**Exercise habits**, ***n*** **(%)**				0.912
Yes	28 (25.5)	13 (23.6)	15 (27.3)	
No	82 (74.5)	42 (76.4)	40 (72.7)	
**Weight-bearing activity**, ***n*** **(%)**				0.619
Yes	105 (95.5)	53 (96.4)	52 (94.5)	
No	5 (4.5)	2 (3.6)	3 (5.5)	
**Fetal head circumference, mm**				0.159
N (Nmiss)	110 (0)	55 (0)	55 (0)	
Mean±SD	33.15 ± 0.62	33.18 ± 0.50	33.13 ± 0.72	
Min–Max	32.0–38.0	32.0–35.5	32–28	
Median (IQR)	33 (0)	33 (0)	33 (0)	
**Supraumbilical IRD, cm^§^**				0.920
N (Nmiss)	110 (0)	55 (0)	55 (0)	
Mean±SD	1.41 ± 1.27	1.26 ± 1.13	1.56 ± 1.39	
Min–Max	0–7	0–3	0–7	
Median (IQR)	1.5 (2.0)	1.0 (2.0)	1.5 (3.0)	
**IRD at the horizontal line of umbilicus, cm**				0.051
N (Nmiss)	110 (0)	55 (0)	55 (0)	
Mean±SD	2.84 ± 0.80	2.74 ± 0.75	2.94 ± 0.85	
Min–Max	1.0–7.0	1.5–5.0	1.0–7.0	
Median (IQR)	3.0 (0.5)	3.0 (1.0)	3.0 (0.5)	
**IRD at the midpoint of the umbilical and pubic symphysis line, cm**				0.654
N (Nmiss)	110 (0)	55 (0)	55 (0)	
Mean±SD	0.18 ± 0.59	0.18 ± 0.62	0.18 ± 0.57	
Min–Max	0–3	0–3	0–3	
Median (IQR)	0 (0)	0 (0)	0 (0)	
**Time to pregnancy, weeks**				0.524
N (Nmiss)	109 (1)	55 (0)	55 (0)	
Mean±SD	38.87 ± 1.31	38.85 ± 1.38	54.85 ± 6.67	
Min–Max	34–42	34–42	42–75	
Median (IQR)	39 (2)	39 (2)	55 (10)	
**Time to postpartum, days**				0.875
N (Nmiss)	110 (0)	55 (0)	55 (0)	
Mean±SD	116.98 ± 78.05	118.07 ± 78.83	115.89 ± 77.97	
Min–Max	43–363	43–346	43–363	
Median (IQR)	87.0 (83.8)	87.0 (77.0)	88 (88)	
**Educational level**, ***n*** **(%)**				0.161
Primary education or less	5 (4.5)	4 (7.3)	1 (1.8)	
Secondary education	9 (8.2)	6 (10.9)	3 (5.5)	
Tertiary education	96 (87.3)	45 (81.8)	51 (92.7)	
**Occupation before this pregnancy**, ***n*** **(%)**				0.797
Yes (including the women who were on sick leave)	107 (97.3)	54 (98.2)	53 (96.4)	
No (homemaker, job seeker or student)	3 (2.7)	1 (1.8)	2 (3.6)	
**Low back pain**, ***n*** **(%)**				0.068
Yes	94 (85.5)	46 (83.6)	48 (87.3)	
No	16 (14.5)	9 (16.4)	7 (12.7)	
**Pelvic girdle pain**, ***n*** **(%)^‡^**	109 (1)	55 (0)	54 (1)	0.792
Yes	59 (53.6)	26 (47.3)	33 (60.0)	
No	50 (45.5)	29 (52.7)	21 (38.2)	
**Urine leakage**, ***n*** **(%)**				0.248
Yes	59 (53.6)	28 (50.9)	31 (56.4)	
No	51 (46.4)	27 (49.1)	24 (43.6)	
**Urinary frequency**, ***n*** **(%)**				0.061
Yes	49 (44.5)	21 (38.2)	28 (50.9)	
No	61 (55.5)	34 (61.8)	27 (49.1)	
**Sexual dysfunction**, ***n*** **(%)**				0.487
Yes	34 (30.9)	15 (27.3)	19 (34.5)	
No	76 (69.1)	40 (72.7)	36 (65.5)	
**Chronic pelvic pain**, ***n*** **(%)**				0.548
Yes	10 (9.1)	5 (9.1)	5 (9.1)	
No	100 (90.9)	50 (90.9)	50 (90.9)	
**Constipation**, ***n*** **(%)**				0.847
Yes	54 (49.1)	28 (50.9)	26 (47.3)	
No	56 (50.9)	27 (49.1)	29 (52.7)	
**Urinary urgency**, ***n*** **(%)**				0.098
Yes	27 (24.5)	16 (29.1)	11 (20.0)	
No	83 (75.5)	39 (70.9)	44 (80.0)	
**Pelvic organ prolapses**, ***n*** **(%)**				0.467
1	42 (38.2)	21 (38.2)	21 (38.2)	
2	65 (59.1)	34 (61.8)	31 (56.4)	
3	3 (2.7)	0	3 (5.5)	
**Supraumbilical AC at supine position, cm^§^**				0.645
N (Nmiss)	110 (0)	55 (0)	55 (0)	
Mean±SD	79.64 ± 6.30	78.27 ± 5.45	81.0 ± 6.82	
Min–Max	64.0–97.5	66.0–95.5	64.0–97.5	
Median (IQR)	79.5 (6.0)	79.0 (4.5)	81.0 (8.7)	
**AC at the horizontal line of umbilicus in supine position, cm**				0.927
N (Nmiss)	110 (0)	55 (0)	55 (0)	
Mean±SD	83.16 ± 6.93	81.56 ± 6.26	84.77 ± 7.24	
Min–Max	66.5–102.8	68.0–96.0	66.5–102.8	
Median (IQR)	83.0 (7.8)	82.8 (6.5)	85.5 (9.8)	
**AC at the midpoint of the umbilical and pubic symphysis line in supine position, cm**				0.726
N (Nmiss)	110 (0)	55 (0)	55 (0)	
Mean±SD	85.39 ± 6.47	83.80 ± 5.59	86.97 ± 6.93	
Min–Max	70–105	72–101	70–105	
Median (IQR)	85.5 (6.9)	84.3 (6.8)	86.0 (9.0)	
**HC at supine position, cm**				0.876
N (Nmiss)	110 (0)	55 (0)	55 (0)	
Mean±SD	91.42 ± 5.58	90.06 ± 5.10	92.78 ± 5.76	
Min**–**Max	78.0**–**106.5	78.0**–**104.7	81.0**–**106.5	
Median (IQR)	91.0 (6.63)	90.0 (5.5)	93.5 (6.5)	
**Supraumbilical AC at standing position, cm^§^**				0.261
N (Nmiss)	85 (25)	44 (11)	41 (14)	
Mean±SD	78.44 ± 6.68	77.28 ± 6.08	79.67 ± 7.14	
Min**–**Max	64.0**–**96.5	64.0**–**94.8	65.0**–**96.5	
Median (IQR)	77.5 (7.9)	77.0 (7.9)	79.0 (8.85)	
**AC at the horizontal line of umbilicus in standing position, cm**				0.509
N (Nmiss)	85 (25)	44 (11)	41 (14)	
Mean±SD	88.38 ± 7.45	87.25 ± 7.14	89.60 ± 7.67	
Min**–**Max	70**–**109	70**–**103	71**–**109	
Median (IQR)	88.0 (9.1)	88.0 (7.8)	89.0 (10.8)	
**AC at the midpoint of the umbilical and pubic symphysis line in standing position, cm**				0.717
N (Nmiss)	85 (25)	44 (11)	41 (14)	
Mean±SD	91.91 ± 6.35	90.43 ± 5.52	93.50 ± 6.84	
Min**–**Max	75.0**–**110.5	79.5**–**106.0	75.0**–**110.5	
Median (IQR)	92.0 (7.5)	90.3 (6.7)	93.5 (8.9)	
**HC at standing position, cm**				0.086
N (Nmiss)	84 (26)	44 (11)	40 (15)	
Mean±SD	93.36 ± 6.06	91.82 ± 5.90	95.07 ± 5.84	
Min**–**Max	78.0**–**111.5	78.0**–**105.5	84.0**–**111.5	
Median (IQR)	93.5 (7.8)	92.3 (6.9)	95.3 (7.3)	
**Abdominal static endurance, s**				0.902
N (Nmiss)	76 (34)	41 (14)	35 (20)	
Mean±SD	8.49 ± 22.16	9.95 ± 26.84	6.77 ± 15.17	
Min**–**Max	0**–**150	0**–**150	0**–**71	
Median (IQR)	0 (4.5)	0 (3)	0 (10)	
**Abdominal dynamic endurance**				0.062
N (Nmiss)	74 (36)	40 (15)	34 (21)	
Mean±SD	3.31 ± 7.03	3.00 ± 6.34	3.68 ± 7.84	
Min–Max	0–28	0–22	0–28	
Median (IQR)	0 (0)	0 (0)	0 (1.75)	
**Left side of umbilics skinfold thickness, mm**				0.667
N (Nmiss)	110 (0)	55 (0)	55 (0)	
Mean±SD	40.48 ± 14.93	39.42 ± 15.30	41.55 ± 14.61	
Min–Max	10–90	11–90	10–74	
Median (IQR)	39.5 (20.0)	36 (23)	40 (20)	
**Right side of umbilics skinfold thickness, mm**				0.974
N (Nmiss)	110 (0)	55 (0)	55 (0)	
Mean±SD	41.84 ± 14.79	40.02 ± 15.45	43.65 ± 14.0	
Min–Max	11–90	11–90	13–73	
Median (IQR)	41.5 (19.3)	37 (17)	44 (17)	
**Right skinfold thickness of triceps brachii, mm**				0.347
N (Nmiss)	81 (29)	42 (13)	39 (16)	
Mean±SD	37.67 ± 9.38	39.21 ± 10.50	36.0 ± 7.79	
Min–Max	15–65	15–65	15–52	
Median (IQR)	38.0 (13.0)	40.0 (16.3)	37.0 (9.0)	
**Right skinfold thickness of scapula, mm**				0.219
N (Nmiss)	81 (29)	42 (13)	39 (16)	
Mean±SD	36.07 ± 10.30	36.93 ± 10.44	35.15 ± 10.21	
Min–Max	15–62	20–62	15–60	
Median (IQR)	35.0 (14.5)	35.5 (15.3)	34.0 (14.0)	
**IRD at the midpoint of umbilicus and xiphoid process in the resting state, cm^Δ^**				0.445
N (Nmiss)	110 (0)	55 (0)	55 (0)	
Mean±SD	22.21 ± 10.83	20.60 ± 7.38	23.81 ± 13.31	
Min–Max	7–97	10–36	7–97	
Median (IQR)	21 (12)	20 (12)	23 (12)	
**IRD at the midpoint of umbilicus and xiphoid process in head-up and flexed knee state, cm^Δ^**				0.544
N (Nmiss)	110 (0)	55 (0)	55 (0)	
Mean±SD	18.13 ± 9.67	17.32 ± 6.88	18.95 ± 11.84	
Min–Max	4–90	5–34	4–90	
Median (IQR)	17 (10)	17 (9)	16 (11)	
**IRD at the midpoint of umbilicus and xiphoid process in end-expiratory state, cm^Δ^**				0.288
N (Nmiss)	110 (0)	55 (0)	55 (0)	
Mean±SD	23.88 ± 11.84	21.95 ± 8.00	25.81 ± 14.55	
Min–Max	7–104	10–40	7–104	
Median (IQR)	22 (14)	22 (13)	24 (15)	
**IRD at the horizontal line of umbilicus in the resting state, cm^Δ^**				0.749
N (Nmiss)	110 (0)	55 (0)	55 (0)	
Mean±SD	36.57 ± 13.73	34.88 ± 9.78	38.26 ± 16.70	
Min–Max	21–114	21–69	21–114	
Median (IQR)	34 (14)	34 (12)	34 (16)	
**IRD at the horizontal line of umbilicus in head-up and flexed knee state, cm^Δ^**				0.540
N (Nmiss)	110 (0)	55 (0)	55 (0)	
Mean±SD	26.94 ± 9.75	26.06 ± 8.67	27.83 ± 10.73	
Min–Max	10–80	14–65	10–80	
Median (IQR)	25.0 (9.3)	25 (9)	25 (9)	
**IRD at the horizontal line of umbilicus in end-expiratory state, cm^Δ^**				0.708
N (Nmiss)	110 (0)	55 (0)	55 (0)	
Mean±SD	38.38 ± 14.69	36.49 ± 11.04	40.27 ± 17.50	
Min–Max	21–118	21–72	22–118	
Median (IQR)	35 (14)	35 (13)	28 (16)	
**IRD at the midpoint of between umbilicus and pubic symphysis, cm^Δ^**				–
N (Nmiss)	110 (0)	55 (0)	55 (0)	
Mean±SD	0	0	0	
Min–Max	0	0	0	
Median (IQR)	0 (0)	0 (0)	0 (0)	
**Left abdominal skinfold, mm^Δ^**				0.454
N (Nmiss)	15 (95)	4 (51)	11 (44)	
Mean±SD	20.47 ± 4.44	20.25 ± 1.50	20.55 ± 5.18	
Min–Max	14–30	19–22	14–30	
Median (IQR)	20.0 (6.0)	20.0 (2.8)	20 (8)	
**Right abdominal skinfold, mm^Δ^**				0.260
N (Nmiss)	15 (95)	4 (51)	11 (44)	
Mean±SD	19.93 ± 3.58	19.75 ± 0.96	20.0 ± 4.20	
Min–Max	15–26	19–21	15–26	
Median (IQR)	19.0 (7.0)	19.5 (1.8)	19 (8)	
**The mean values of pre-baseline at the period of calm**				0.043
N (Nmiss)	109 (1)	55 (0)	54 (1)	
Mean±SD	7.10 ± 3.91	7.02 ± 4.43	7.18 ± 3.35	
Min–Max	0.58–23.16	1.14–23.16	0.28–14.91	
Median (IQR)	6.2 (5.1)	5.7 (5.8)	6.81 (4.54)	
**The mean values of fast muscle at the period of systolic**				0.427
N (Nmiss)	109 (1)	55 (0)	54 (1)	
Mean±SD	27.88 ± 11.42	28.55 ± 10.37	27.20 ± 12.46	
Min–Max	5.09–49.61	9.12–48.83	5.09–49.61	
Median (IQR)	28.2 (18.2)	28.5 (17.8)	27.1 (20.66)	
**The mean values of comprehensive muscle at the period of systolic**				0.005
N (Nmiss)	109 (1)	55 (0)	54 (1)	
Mean±SD	20.74 ± 10.74	20.48 ± 10.20	21.01 ± 11.35	
Min–Max	3.16–43.76	3.19–40.84	3.16–43.76	
Median (IQR)	18.6 (17.2)	18.2 (17.1)	18.7 (18.42)	
**The mean values of slow muscle at the period of systolic**				0.001
N (Nmiss)	109 (1)	55 (0)	54 (1)	
Mean±SD	18.13 ± 9.72	17.40 ± 9.06	18.87 ± 10.39	
Min–Max	2.85**–**40.50	2.85**–**40.50	4.12**–**38.97	
Median (IQR)	16.4 (14.2)	16.1 (13.3)	16.61 (15.77)	
**The mean values of post-baseline at the period of calm**				0.225
N (Nmiss)	109 (1)	55 (0)	54 (1)	
Mean±SD	6.47 ± 3.78	6.24 ± 3.66	6.70 ± 3.69	
Min–Max	0.58**–**19.12	1.21**–**19.12	0.58**–**16.52	
Median (IQR)	6.5 (5.2)	5.8 (4.3)	6.6 (4.9)	
**Leeds dyspepsia questionnaire**				0.716
N (Nmiss)	110	55	55	
Mean±SD	6.55 ± 1.22	6.49 ± 1.09	6.60 ± 1.34	
Min–Max	6**–**13	6**–**12	6**–**13	
Median (IQR)	6.0 (0.25)	6.5 (0)	6.0 (1)	
**SAT in the paraumbilical region**				0.069
N (Nmiss)	110 (0)	55 (0)	55 (0)	
Mean±SD	41.16 ± 14.84	39.38 ± 15.38	43.01 ± 14.09	
Min–Max	10**–**90	11**–**90	10**–**74	
Median (IQR)	40 (20)	36 (20)	43 (18.5)	
**SAT in right triceps region**				0.124
N (Nmiss)	81 (29)	41 (14)	40 (15)	
Mean±SD	37.67 ± 9.38	39.21 ± 10.50	36 ± 7.79	
Min–Max	15**–**65	15**–**65	15**–**52	
Median (IQR)	38 (13)	40 (16.25)	37 (9)	
**SAT in the right subscapular region**				0.442
N (Nmiss)	81 (29)	41 (14)	40 (15)	
Mean±SD	36.07 ± 10.31	36.93 ± 10.44	35.15 ± 10.21	
Min–Max	15**–**62	20**–**62	15**–**60	
Median (IQR)	35 (14.5)	35.5 (15.25)	34 (14)	
**F%**				0.380
N (Nmiss)	110 (0)	55 (0)	55 (0)	
Mean±SD	0.32 ± 0.20	0.34 ± 0.21	0.30 ± 0.19	
Min–Max	0.02**–**0.73	0.02**–**0.73	0.02**–**0.62	
Median (IQR)	0.37 (0.44)	0.39 (0.40)	0.35 (0.41)	

AC, abdominal circumference; EA, electroacupuncture; BMI, body mass index; SD, Standard Deviation; HC, hip circumference; IQR, Inter-Quartile Range; IRD, inter recti distance.

* The only one patient (1 [who withdrew with low back pain] in control group) did not receive the study consecutive treatment. The follow-up to 26 weeks was incomplete for 3 patients (Due to the COVID-19, there is no way to follow up on time in other places).

† There were no significant differences between two groups.

‡ The pelvic girdle includes inguinal, pubic symphysis, coccyx, sacrum, and either side of the pelvis.

§ The midpoint of umbilicus and xiphoid process.

Δ At the supine position.

Briefly, EA and control groups were comparable with respect to demographic characteristics at baseline (Table 2). Minor adverse events (bruising and bleeding from sites of needle insertion) occurred in five (9.1%) patients from the EA group (Supplementary material 2), and minor adverse events (a little lumbar acid) occurred in thirteen (23.64%) control group patients (Supplementary material 2). There were no serious adverse events that were attributed to the study intervention in either group.

### Blinding assessments

Outcome assessors and physical therapists (PT) responded to the assessment of blinding questions at week 2. Statisticians responded to the assessment of blinding questions at week 26 (Table 3). For the three categories of responders, the majority reported: “don't know". The PT had six (10.91%) accurate guesses for the EA group, and three (5.45%) correct guesses were for the control group. For the Bang index where values between −0.2 and 0.2 indicate successful blinding, values for the EA group and control group were 0 and 0, respectively for outcome assessors. For the PT, the Bang index values were 0.109 (95% CI = 0.031–0.187) for the EA group, and 0.115 (95% CI = −0.004 to 0.114) for the control group. For statisticians, the Bang index values were 0 for the EA group, and 0 for the control group. James' Blinding index (BI) assesses the overall degree of disagreement between treatment allocation and guess, where BI < 0.5 represents unblinding. James' Blinding index (BI) was 1, 0.959 (95% CI = 0.920–0.998), and 1, respectively, for outcome assessors, physical therapists (PT), and statisticians. Blinding index values suggest that blinding was achieved for outcome assessors, physical therapists (PT), and statisticians.

**Table 3:** Guesstimate vs. treatment assignment.

**Treatment assignment**	**Outcome assessors (*n =* 110)**		**Physical therapists (*n =* 110)**		**Statisticians (*n =* 110)**	
	**EA group**	**Control group**	**EA group**	**Control group**	**EA group**	**Control group**
**Guesstimate^a^**						
EA group	0	0	6 (10.91)	3 (5.45)	0	0
Control group	0	0	0	0	0	0
Don't know	55	55	49 (89.09)	52 (94.55)	55	55
**Degree of confidence in response^a^**						
**Correct guesstimate**						
Extremely confident	0	0	0	0	0	0
Reasonably confident	0	0	0	1	0	0
Slightly confident	0	0	6 (10.91)	2	0	0
Missing	55	55	49 (89.09)	52 (94.55)	55	55
**Incorrect guesstimate**						
Extremely confident	0	0	0	0	0	0
Reasonably confident	0	0	0	0	0	0
Slightly confident	0	0	0	0	0	0
Missing	0	0	0	0	0	0
**Blinding indices**						
James' Blinding Index^b^	1		0.959 (0.920, 0.998)		1	
**Bang Blinding Index^b^**						
EA group	0		0.109 (0.031, 0.187)		0	
Control group	0		0.115 (−0.004, 0.114)		0	

James' Blinding index (BI) assesses overall degree of disagreement between treatment allocation and guess, where a BI < 0.5 represents unblinding.

Bang Blinding index assesses the degree of disagreement in each treatment group, where a BI>0.2 represents unblinding and a BI < -0.2 represents Opposite Guess or ‘Wishful thinking'.

a Parentheses denote percentages.

b Parentheses denote 95% confidence interval.

At 2 weeks, the mean of IRD at the horizontal line of the umbilicus, the midpoint of the umbilicus, and the xiphoid process in all states of the two groups were reduced compared with those before the treatment, with statistical significance (*P* < 0.05). For the difference of IRD at the horizontal line of the umbilicus in end-expiratory state, the EA group was better than the control group, with a statistically significant *P* < 0.05. The mean of IRD at the horizontal line of the umbilicus in the end-expiratory state was smaller in the EA group than in the control group, but the difference was not statistically significant (*P* > 0.05) (Table 4).

**Table 4:** Primary and secondary outcomes^*^.

**Outcome**		**EA group** ** (*n =* 54)**	**Control group** ** (*n =* 52)**	***P*-value^##^**
**Primary outcome**				
**IRD**				
IRD at the horizontal line of umbilicus in the resting state, cm ^Δ^^†^	Before the treatment	3.49 ± 0.98	3.83 ± 1.67	
	After the treatment	2.85 ± 0.86	3.08 ± 1.43	
	*P-*value^#^	0.000	0.000	
IRD at the horizontal line of umbilicus in head-up and flexed knee state, cm ^Δ^^†^	Before the treatment	2.61 ± 0.87	2.78 ± 1.07	
	After the treatment	1.96 ± 0.61	2.17 ± 0.81	
	*P-*value ^#^	0.000	0.000	
IRD at the horizontal line of umbilicus in end-expiratory state, cm ^Δ^^†^	Before the treatment	3.65 ± 1.10	4.03 ± 1.75	
	After the treatment	3.21 ± 0.96	3.43 ± 1.57	
	*P-*value ^#^	0.000	0.000	
IRD at the midpoint of umbilicus and xiphoid process in the resting state, cm ^Δ^^†^	Before the treatment	2.06 ± 0.74	2.38 ± 1.33	
	After the treatment	1.60 ± 0.72	1.82 ± 1.24	
	*P-*value ^#^	0.000	0.000	
IRD at the midpoint of umbilicus and xiphoid process in head-up and flexed knee state, cm ^Δ^^†^	Before the treatment	1.73 ± 0.69	1.90 ± 1.18	
	After the treatment	1.33 ± 0.62	1.41 ± 0.80	
	*P-*value ^#^	0.000	0.000	
IRD at the midpoint of umbilicus and xiphoid process in end-expiratory state, cm ^Δ^^†^	Before the treatment	2.20 ± 0.80	2.58 ± 1.46	
	After the treatment	1.76 ± 0.78	2.03 ± 1.39	
	*P-*value ^#^	0.000	0.000	
IRD at the midpoint of between umbilicus and pubic symphysis, cm ^Δ^^†^	Before the treatment	0.00 ± 0.00	0.00 ± 0.00	
	After the treatment	0.00 ± 0.00	0.00 ± 0.00	
	*P-*value ^#^	1.000	1.000	
IRD at the horizontal line of umbilicus in the resting state, cm ^Δ^^†^	After the treatment	2.85 ± 0.86	3.08 ± 1.43	
	At 24 weeks follow-up after treatment	2.59 ± 0.84	2.89 ± 1.31	
	*P-*value ^#^	0.000	0.000	
IRD at the horizontal line of umbilicus in head-up and flexed knee state, cm ^Δ^^†^	After the treatment	1.96 ± 0.6	2.17 ± 0.81	
	At 24 weeks follow-up after treatment	1.77 ± 0.67	2.08 ± 0.82	
	*P-*value ^#^	0.167	0.001	
IRD at the horizontal line of umbilicus in end-expiratory state, cm ^Δ^^†^	After the treatment	3.21 ± 0.96	3.43 ± 1.57	
	At 24 weeks follow-up after treatment	3.00 ± 1.02	3.25 ± 1.30	
	*P-*value ^#^	0.001	0.000	
IRD at the midpoint of umbilicus and xiphoid process in the resting state, cm ^Δ^^†^	After the treatment	1.60 ± 0.72	1.82 ± 1.24	
	At 24 weeks follow-up after treatment	1.37 ± 0.72	1.50 ± 1.24	
	*P-*value ^#^	0.000	0.000	
IRD at the midpoint of umbilicus and xiphoid process in head-up and flexed knee state, cm ^Δ^^†^	After the treatment	1.33 ± 0.62	1.41 ± 0.80	
	At 24 weeks follow-up after treatment	1.12 ± 0.63	1.17 ± 0.74	
	*P-*value ^#^	0.000	0.000	
IRD at the midpoint of umbilicus and xiphoid process in end-expiratory state, cm ^Δ^^†^	After the treatment	1.76 ± 0.78	2.03 ± 1.39	
	At 24 weeks follow-up after treatment	1.56 ± 0.83	1.68 ± 1.39	
	*P-*value ^#^	0.000	0.000	
IRD at the midpoint of between umbilicus and pubic symphysis, cm ^Δ^^†^	After the treatment	0.00 ± 0.00	0.00 ± 0.00	
	At 24 weeks follow-up after treatment	0.00 ± 0.00	0.00 ± 0.00	
	*P-*value ^#^	1.000	1.000	
**Week 2^##^**
IRD at the horizontal line of umbilicus in the resting state, cm ^Δ^^‡^		−7.56 ± 3.82	−6.37 ± 3.67	0.084
IRD at the horizontal line of umbilicus in head-up and flexed knee state, cm ^Δ^^‡^		−6.20 ± 5.37	−6.48 ± 5.05	0.884
IRD at the horizontal line of umbilicus in end-expiratory state, cm ^Δ^^‡^		−6.09 ± 3.91	−4.44 ± 4.62	0.017
IRD at the midpoint of umbilicus and xiphoid process in the resting state, cm ^Δ^^‡^		−5.58 ± 3.24	−4.64 ± 2.11	0.212
IRD at the midpoint of umbilicus and xiphoid process in head-up and flexed knee state, cm ^Δ^^‡^		−4.89 ± 5.89	−4.06 ± 3.62	0.472
IRD at the midpoint of umbilicus and xiphoid process in end-expiratory state, cm ^Δ^^‡^		−5.43 ± 3.94	−4.32 ± 2.74	0.128
IRD at the midpoint of between umbilicus and pubic symphysis, cm ^Δ^^‡^		0 ± 0	0 ± 0	1.000
**Week 26^##^**				
IRD at the horizontal line of umbilicus in the resting state, cm ^Δ^^‡^		−2.57 ± 3.12	−2.17 ± 3.31	0.361
IRD at the horizontal line of umbilicus in head-up and flexed knee state, cm ^Δ^^‡^		−1.94 ± 4.22	−0.88 ± 4.01	0.146
IRD at the horizontal line of umbilicus in end-expiratory state, cm ^Δ^^‡^		−1.98 ± 4.27	−2.08 ± 4.54	0.429
IRD at the midpoint of umbilicus and xiphoid process in the resting state, cm ^Δ^^‡^		−2.37 ± 2.08	−3.42 ± 3.31	0.153
IRD at the midpoint of umbilicus and xiphoid process in head-up and flexed knee state, cm ^Δ^^‡^		−2.09 ± 2.58	−2.48 ± 3.46	0.932
IRD at the midpoint of umbilicus and xiphoid process in end-expiratory state, cm ^Δ^^‡^		−2.17 ± 2.89	−3.69 ± 3.91	0.056
IRD at the midpoint of between umbilicus and pubic symphysis, cm ^Δ^^‡^		0 ± 0	0 ± 0	1.000
**Week 2^##^**				
IRD at the horizontal line of umbilicus in the resting state, cm ^Δ^^†^		2.85 ± 0.86	3.08 ± 1.43	0.736
IRD at the horizontal line of umbilicus in head-up and flexed knee state, cm ^Δ^^†^		1.96 ± 0.61	2.17 ± 0.81	0.194
IRD at the horizontal line of umbilicus in end-expiratory state, cm ^Δ^^†^		3.21 ± 0.96	3.43 ± 1.57	0.851
IRD at the midpoint of umbilicus and xiphoid process in the resting state, cm ^Δ^^†^		1.60 ± 0.72	1.82 ± 1.24	0.401
IRD at the midpoint of umbilicus and xiphoid process in head-up and flexed knee state, cm ^Δ^^†^		1.33 ± 0.62	1.41 ± 0.80	0.593
IRD at the midpoint of umbilicus and xiphoid process in end-expiratory state, cm ^Δ^^†^		1.76 ± 0.78	2.03 ± 1.39	0.338
IRD at the midpoint of between umbilicus and pubic symphysis, cm ^Δ^^†^		0.00 ± 0.00	0.00 ± 0.00	1.000
**Week 26^##^**				
IRD at the horizontal line of umbilicus in the resting state, cm ^Δ^^†^		2.59 ± 0.84	2.89 ± 1.31	0.224
IRD at the horizontal line of umbilicus in head-up and flexed knee state, cm ^Δ^^†^		1.77 ± 0.67	2.08 ± 0.82	0.027
IRD at the horizontal line of umbilicus in end-expiratory state, cm ^Δ^^†^		3.00 ± 1.02	3.25 ± 1.30	0.450
IRD at the midpoint of umbilicus and xiphoid process in the resting state, cm ^Δ^^†^		1.37 ± 0.72	1.50 ± 1.24	0.704
IRD at the midpoint of umbilicus and xiphoid process in head-up and flexed knee state, cm ^Δ^^†^		1.12 ± 0.63	1.17 ± 0.74	0.562
IRD at the midpoint of umbilicus and xiphoid process in end-expiratory state, cm ^Δ^^†^		1.56 ± 0.83	1.68 ± 1.39	0.756
IRD at the midpoint of between umbilicus and pubic symphysis, cm ^Δ^^†^		0 ± 0	0 ± 0	1.000
Electromyographic evaluation of pelvic floor				
**Week 2^#^**
The mean value of pre-baseline during the period of calm^†^	Before the treatment	7.02 ± 4.43	7.17 ± 3.32	
	After the treatment	5.55 ± 3.58	6.56 ± 3.64	
	*P-*value ^#^	0.000	0.050	
Fast muscle during systole^†^	Before the treatment	28.55 ± 10.37	27.37 ± 12.40	
	After the treatment	35.41 ± 10.59	32.86 ± 12.48	
	*P-*value ^#^	0.000	0.000	
The comprehensive muscle during systole^†^	Before the treatment	20.48 ± 10.20	20.48 ± 10.20	
	After the treatment	27.33 ± 10.38	25.86 ± 10.52	
	*P-*value ^#^	0.000	0.000	
Slow muscle during systole^†^	Before the treatment	17.40 ± 9.06	18.77 ± 10.32	
	After the treatment	24.82 ± 9.70	23.54 ± 0.81	
	*P-*value ^#^	0.000	0.000	
The mean value of post-baseline during the period of calm^†^	Before the treatment	6.24 ± 3.88	6.70 ± 3.65	
	After the treatment	6.76 ± 4.18	7.37 ± 3.70	
	*P-*value ^#^	0.463	0.149	
**Week 26^#^**
The mean value of pre-baseline during the period of calm^†^	Before the treatment	5.55 ± 3.58	6.56 ± 3.64	
	After the treatment	4.44 ± 2.29	6.25 ± 3.87	
	*P-*value ^#^	0.004	0.348	
Fast muscle during systole^†^	Before the treatment	35.41 ± 10.59	32.86 ± 12.48	
	After the treatment	46.08 ± 14.91	39.64 ± 19.76	
	*P-*value ^#^	0.000	0.008	
The comprehensive muscle during systole^†^	Before the treatment	27.33 ± 10.38	25.86 ± 10.52	
	After the treatment	39.64 ± 19.76	28.67 ± 16.07	
	*P-*value ^#^	0.002	0.120	
Slow muscle during systole	Before the treatment	24.82 ± 9.70	23.54 ± 0.81	
	After the treatment	29.10 ± 10.80	23.02 ± 11.22	
	*P-*value ^#^	0.003	0.579	
The mean value of post-baseline during the period of calm^†^	Before the treatment	6.76 ± 4.18	7.37 ± 3.70	
	After the treatment	5.95 ± 2.55	7.38 ± 5.31	
	*P-*value ^#^	0.213	0.289	
**Week 2^##^**
The mean value of pre-baseline during the period of calm^‡^		−1.47 ± 3.43	−0.64 ± 2.90	0.225
The mean value of fast muscle during systole^‡^		6.86 ± 7.50	5.59 ± 9.51	0.178
The mean value of the comprehensive muscle during systole^‡^		6.86 ± 7.14	4.87 ± 7.82	0.074
The mean value of slow muscle during systole^‡^		7.42 ± 6.39	4.63 ± 8.21	0.019
The mean value of post-baseline during the period of calm^‡^		0.51 ± 3.32	0.62 ± 2.93	0.630
**Week 26^##^**
The mean value of pre-baseline during the period of calm^‡^		−1.21 ± 2.74	−0.41 ± 3.54	0.355
The mean value of fast muscle during systole^‡^		10.36 ± 12.7	7.07 ± 16.02	0.102
The mean value of the comprehensive muscle during systole^‡^		5.54 ± 11.77	3.05 ± 12.66	0.191
The mean value of slow muscle during systole^‡^		4.03 ± 9.30	−0.19 ± 9.29	0.013
The mean value of post-baseline during the period of calm^‡^		−0.91 ± 3.44	0.01 ± 4.75	0.970
**Week 2^##^**
The mean value of pre-baseline during the period of calm^†^		5.55 ± 3.58	6.56 ± 3.64	0.116
The mean value of fast muscle during systole^†^		35.41 ± 10.59	32.86 ± 12.48	0.212
The mean value of the comprehensive muscle during systole^†^		27.33 ± 10.38	25.86 ± 10.52	0.486
The mean value of slow muscle during systole^†^		24.82 ± 9.70	23.54 ± 0.81	0.575
The mean value of the mean value of post-baseline during the period of calm^†^		6.76 ± 4.18	7.37 ± 3.70	0.211
**Week 26^##^**
The mean value of pre-baseline during the period of calm^†^		4.44 ± 2.29	6.25 ± 3.87	0.006
The mean value of fast muscle during systole^†^		46.08 ± 14.91	39.64 ± 19.76	0.006
The mean value of the comprehensive muscle during systole^†^		33.15 ± 12.66	28.67 ± 16.07	0.016
The mean value of slow muscle during systole^†^		29.10 ± 10.80	23.02 ± 11.22	0.002
The mean value of post-baseline during the period of calm^†^		5.95 ± 2.55	7.38 ± 5.31	0.235
**Secondary Outcomes**
The elastic of linea alba				
**Week 2^##^**
The elastic of linea alba in the horizontal line of umbilicus		3.08 ± 0.43	2.24 ± 0.74	0.000
The elastic of linea alba in the midpoint of umbilicus and xiphoid process		2.34 ± 0.65	1.24 ± 0.48	0.000
**Week 26^##^**
The elastic of linea alba in the horizontal line of umbilicus		3.94 ± 0.72	3.16 ± 0.93	0.000
The elastic of linea alba in the midpoint of umbilicus and xiphoid process		3.23 ± 0.85	2.72 ± 1.01	0.010
BMI at week 2		21.97 ± 0.05	23.25 ± 0.42	0.013
The paraumbilical SAT				
SAT in the paraumbilical region^†^	Before the treatment	39.38 ± 15.38	43.01 ± 14.09	
	After the treatment	35.02 ± 11.97	37.85 ± 12.05	
	*P-*value ^#^	0.000	0.000	
SAT in right triceps region^†^	Before the treatment	39.21 ± 10.50	36 ± 7.79	
	After the treatment	34.58 ± 7.03	33.04 ± 6.65	
	*P-*value^#^	0.001	0.024	
SAT in the right subscapular region^†^	Before the treatment	36.93 ± 10.44	35.15 ± 10.21	
	After the treatment	31.64 ± 7.51	33.62 ± 8.56	
	*P-*value^#^	0.000	0.604	
*F*% ^†^	Before the treatment	0.34 ± 0.21	0.30 ± 0.19	
	After the treatment	0.31 ± 0.16	0.34 ± 0.14	
	*P-*value^#^	0.142	0.067	
SAT in the paraumbilical region^†^	After the treatment	35.02 ± 11.97	37.85 ± 12.05	
	At 24 weeks follow-up after treatment	31.64 ± 7.51	31.77 ± 8.83	
	*P-*value^#^	0.000	0.000	
SAT in right triceps region^†^	After the treatment	34.58 ± 7.03	33.04 ± 6.65	
	At 24 weeks follow-up after treatment	28.89 ± 7.26	30.08 ± 5.97	
	*P-*value^#^	0.002	0.040	
SAT in the right subscapular region^†^	After the treatment	31.64 ± 7.51	33.62 ± 8.56	
	At 24 weeks follow-up after treatment	32.22 ± 9.05	31.32 ± 8.95	
	*P-*value^#^	0.218	0.216	
*F*% ^†^	After the treatment	0.31 ± 0.16	0.34 ± 0.14	
	At 24 weeks follow-up after treatment	0.34 ± 0.10	0.33 ± 0.11	
	*P-*value^#^	0.246	0.723	
**Week 2^##^**				
SAT in the paraumbilical region^†^		35.02 ± 11.97	37.85 ± 12.05	0.084
SAT in right triceps region^†^		34.58 ± 7.03	33.04 ± 6.65	0.285
SAT in the right subscapular region^†^		31.64 ± 7.51	33.62 ± 8.56	0.244
F% ^†^		0.31 ± 0.16	0.34 ± 0.14	0.362
SAT in the paraumbilical region^‡^		−5.60 ± 9.93	−5.16 ± 9.03	0.726
SAT in right triceps region^‡^		−0.81 ± 9.33	1.38 ± 9.43	0.084
SAT in the right subscapular region^‡^		−1.13 ± 8.44	1.94 ± 9.00	0.010
F% ^‡^		−0.01 ± 0.10	0.02 ± 0.09	0.019
**Week 26^##^**				
SAT in the paraumbilical region^†^		29.23 ± 8.66	31.77 ± 8.83	0.038
SAT in right triceps region^†^		28.89 ± 7.26	30.08 ± 5.97	0.365
SAT in the right subscapular region^†^		32.22 ± 9.05	31.32 ± 8.95	0.611
F% ^†^		0.34 ± 0.10	0.33 ± 0.11	0.586
SAT in the paraumbilical region^‡^		−11.19 ± 19.44	−13.59 ± 17.12	0.332
SAT in right triceps region^‡^		−0.78 ± 15.38	0.93 ± 13.86	0.390
SAT in the right subscapular region^‡^		1.69 ± 14.74	1.81 ± 13.79	0.951
F% ^‡^		0.00 ± 0.16	0.01 ± 0.15	0.599

AC, abdominal circumference; EA, electroacupuncture; BMI, body mass index; SD, Standard Deviation; HC, hip circumference; IQR, Inter-Quartile Range; IRD, inter recti distance; BMI, Body Mass Index; SAT, subcutaneous adipose tissue; F%, percentage body fat.

* Data for 106 patients (54 randomized to the EA group and 52 to the control group) were used in the final analysis.

# Comparisons of means within group.

## Comparisons were carried out between groups.

† The mean value.

‡ The difference.

§ The midpoint of umbilicus and xiphoid process.

Δ At the supine position.

At 26 weeks follow-up, the mean of IRD at all status in the midpoint of umbilicus and xiphoid process, at the horizontal line of umbilicus in the resting state, and the horizontal line of umbilicus in the end-expiratory state in both groups were reduced compared with those at 26 weeks, and the difference was statistically significant (*P* < 0.05). The mean of IRD at the horizontal line of the umbilicus in head-up and flexed knee state was smaller in the EA group than in the control group, and the difference was statistically significant (*P* < 0.05). The IRD difference at the horizontal line of the umbilicus in head-up and flexed knee state was higher in the EA group than in the control group, but the difference was not statistically significant (*P* > 0.05). The between-group differences in the mean change from baseline in the IRD followed similar trends of stabilizing during follow-up (Table 4).

The results of the electromyographic evaluation of the pelvic floor show the following: After treatment, the mean of pre-baseline during the period of calm in both groups was lower than that before treatment, and the difference was statistically significant (*P* < 0.05). The mean value of the fast muscle during systole, the comprehensive muscle during systole, and the slow muscle during systole in both groups increased compared with that before treatment, and the difference was statistically significant (*P* < 0.05). The difference in slow muscle during systole before and after treatment in the EA group was higher than that in the control group, and the difference was statistically significant (*P* < 0.05). After 26 weeks of follow-up, the mean of pre-baseline during the period of calm in the EA group was lower than those after treatment, and the difference was statistically significant (*P* < 0.05). The mean of the fast muscle during systole, the comprehensive muscle during systole, and the slow muscle during systole in the EA group were increased compared with that after treatment, and the difference was statistically significant (*P* < 0.05). At 26 weeks, the differences in the mean of slow muscle during systole were higher in the EA group than in the control group, and the difference was statistically significant (*P* < 0.05). At 26 weeks, the mean of the pre-baseline during the period of calm of the EA group was lower than that of the control group, and the difference was statistically significant (*P* < 0.05). Compared with the control group, the mean of the fast muscle during systole, the comprehensive muscle during systole, and the slow muscle during systole in the EA group were increased, and the differences were statistically significant (*P* < 0.05) (Table 4).

In the control group, the elasticity of linea alba was smaller than that of the EA group at two sites (the horizontal line of the umbilicus, and the midpoint of the umbilicus and xiphoid process) at week 2 and week 26 (*P* < 0.05). In terms of the correlation between the elasticity of linea alba and IRD, the LA elasticity score was negatively correlated with IRD (rs = −0.356, *P* < 0.05). As recognized by week 2, a greater decrease in BMI in the EA group compared with the control group indicate the presence of variation in response to treatment (*P* < 0.05) (Table 4).

After treatment, the mean of SAT at the paraumbilical and right triceps of the two groups, and the mean of SAT at the right subscapular of the EA group were reduced compared with those before treatment, with statistical significance (*P* < 0.05), but the difference was not statistically significant (*P* > 0.05) between groups. The F% difference and the right subscapular SAT were reduced in the EA group than in the control group on the front-to-back difference between groups, with statistical significance (*P* < 0.05) (Table 4). The comparison within the group suggested that the total LDQ score of the EA group improved compared with that before treatment and was statistically significant (*P* < 0.05). However, the difference between the control group after and before treatment was not statistically different (*P* > 0.05), and the comparison between the groups suggested that the total LDQ score after treatment was not statistically different between the two groups (*P* > 0.05). At 26 weeks follow-up, the intra-group comparison suggested that the difference in the total LDQ scores between the EA group after follow-up and before treatment improved and was statistically significant (*P* < 0.05), and the difference between the EA group after follow-up and after treatment was not statistically significant (*P* > 0.05). Comparisons between groups suggested no statistical difference (*P* > 0.05). At 26 weeks, 38 in the control group and 39 in the EA group had menstruated. Comparison between the groups suggested no significant difference in menstrual symptoms between the two groups. At follow-up, a comparison between groups suggested that the EA group had better PF than the control group, which was statistically significant (*P* < 0.05). No statistically significant differences were seen in the remaining dimensions. The intra-group comparisons suggested that the SF-MPQ total score and entry change values for the low back at that time were significantly better in both groups after treatment than before treatment, and inter-group comparisons suggested that there was no statistically significant difference (*P* < 0.05) in the SF-MPQ total score and entry change values for the low back after treatment in both groups. The intra-group comparison suggested a statistical difference in the total EPDS score between the two groups (*P* < 0.05), but the inter-group comparison suggested no statistical difference in the total EPDS score between the two groups (*P* > 0.05) (Table 5).

**Table 5:** Other outcomes^*^.

**Outcome**		**EA group** ** (*n =* 54)**	**Control group** ** (*n =* 52)**	***P-*value^##^**
**LDQ**				
Week 2^†^		6.34 ± 0.14	6.29 ± 0.12	0.840
	*P-*value^#^	0.005	0.300	
Week 26^†^		6.11 ± 0.07	6.65 ± 0.25	0.057
	*P-*value^#^	0.134	0.147	
	*P-*value of difference between post-follow-up and pre-treatment^#^	0.017	0.548	
	*P-*value of difference between post-follow-up and post-treatment^#^	0.134	0.147	
	upper abdominal pain	1 ± 0	1.06 ± 0.05	0.147
	postprandial fullness	1.02 ± 0.02	1.16 ± 0.07	0.081
	early satiety	1.04 ± 0.04	1.08 ± 0.05	0.301
	upper abdominal cauterization	1 ± 0	1.04 ± 0.03	0.147
	postprandial nausea	1.02 ± 0.02	1.04 ± 0.03	0.537
	belching	1.04 ± 0.03	1.27 ± 0.11	0.020
**Menstrual symptoms**				
Week 26		10.97 ± 0.59	20.15 ± 0.60	0.801
**QoL**
Week 26		6.34 ± 0.14	6.29 ± 0.12	0.840
PF^†^.		95.19 ± 1.03	92.84 ± 1.19	0.025
PF^‡^		12.17 ± 2.08	12.35 ± 1.79	0.946
RP^†^		96.23 ± 2.22	96.08 ± 1.66	0.492
RP^‡^		41.04 ± 6.43	25 ± 5.22	0.062
BP^†^		87.13 ± 1.79	88.82 ± 1.65	0.500
BP^‡^		16.77 ± 2.57	15.86 ± 1.95	0.770
GH^†^		76.26 ± 2.60	72.33 ± 3.09	0.410
GH^‡^		13.43 ± 3	9.53 ± 2.28	0.291
VT^†^		78.11 ± 2.10	78.24 ± 1.74	0.865
VT^‡^		13.21 ± 2.36	8.82 ± 2.62	0.134
SF^†^		116.75 ± 2.09	114.71 ± 2.29	0.242
SF^‡^		12.97 ± 3.16	11.52 ± 2.93	0.529
RE^†^		89.31 ± 3.15	93.46 ± 2.15	0.456
RE^‡^		28.3 ± 6.19	28.76 ± 5.82	0.936
MH^†^		76.53 ± 2.18	75.61 ± 1.83	0.722
MH^‡^		7.7 ± 2.53	6.82 ± 2.31	0.794
**SF-MPQ**				
Week 2- Pain rating Index (PRI)^‡^		1.38 ± 0.23	1.22 ± 0.20	0.562
	*P-*value^#^	0.000	0.000	
Week 2- sensory item scores^‡^		0.85 ± 0.15	0.86 ± 0.15	0.997
	*P-*value^#^	0.000	0.000	
Week 2- emotional item scores^‡^		0.53 ± 0.12	0.35 ± 0.11	0.181
	*P-*value^#^	0.000	0.000	
Week 2-VAS scores^‡^		0.87 ± 0.58	1.00 ± 0.16	0.587
	*P-*value^#^	0.000	0.000	
Week 2- present pain intensity (PPI) ^‡^		1.17 ± 0.05	1.33 ± 0.07	0.055
	*P-*value^#^	0.000	0.001	
Week 26				
Week 26- Pain rating Index (PRI)^‡^		1.04 ± 0.17	1.49 ± 0.27	0.322
	*P-*value^#^	0.129	0.307	
Throbbing pain		0	0.08 ± 0.04	0.039
Tingling		0	0.02 ± 0.02	0.308
Cutting pains		0	0	1
Sharp pain		0	0	1
Spasmodic pain		0	0	1
Biting pain		0	0	1
Burning pain		0	0	1
Soreness		0.55 ± 0.08	0.69 ± 0.10	0.311
Cramping and swelling pain		0.04 ± 0.03	0.14 ± 0.06	0.125
Tender		0	0.02 ± 0.02	0.308
Cleavage pain		0	0.04 ± 0.04	0.308
Week 26-sensory scores^‡^		0.58 ± 0.10	0.98 ± 0.18	0.185
	*P-*value^#^	0.125	0.468	
Week 26- emotional item scores^‡^		0.45 ± 0.13	0.51 ± 0.12	0.642
	*P-*value^#^	0.502	0.202	
Week 26- VAS scores^‡^		0.57 ± 0.09	1.12 ± 0.17	0.051
	*P-*value^#^	0.035	0.472	
Week 26- present pain intensity (PPI) ^‡^		1.06 ± 0.03	1.16 ± 0.06	0.160
	*P-*value^#^	0.058	0.039	
**EPDS**				
Week 2^†^		6.53 ± 0.58	5.80 ± 0.52	0.455
	*P-*value^#^	0.005	0.002	
Week 26^†^		5.3 ± 0.65	6 ± 0.76	0.475
	*P-*value^#^	0.001	0.000	
Week 2^‡^		−1.23 ± 0.57	0.20 ± 0.77	0.222
	*P-*value^#^	0.319	0.101	
Week 26^‡^		−2.64 ± 0.66	−1.67 ± 0.76	0.469
	*P-*value of difference between post-follow-up and pre-treatment^#^	0.001	0.004	
	*P-*value of difference between post-follow-up and post-treatment^#^	0.029	0.773	
**MBIS**				
Week 2		16.39 ± 5.60	14.94 ± 4.55	0.328
	*P-*value^#^	0.002	0.050	
Week 26		14.21 ± 6.03	13.02 ± 5.57	0.554
	*P-*value^#^	0.001	0.001	
**HerQles**				
Week 2		15.96 ± 10.25	14.24 ± 10.89	0.267
	*P-*value^#^	0.000	0.000	
Week 26		13.36 ± 4.35	12.96 ± 4.96	0.824
	*P-*value^#^	0.001	0.001	
**DRA-related symptom**				
**Urine leakage**				
Week 2		5 (9.3%)	9 (16.7%)	0.29
Week 26		7 (13.0%)	14 (26.9%)	0.104
**Urinary frequency**				
Week 2		4 (7.4%)	4 (7.4%)	0.74
Week 26		0	3 (5.8%)	0.072
**Urinary urgency**				
Week 2		3 (5.6%)	2 (3.7%)	0.33
Week 26		2 (3.7%)	1 (1.9%)	0.594
**Constipation**				
Week 2		12(0.22%)	7 (13.0%)	0.22
Week 26		5 (9.3%)	7 (13.5%)	0.677
**Sexual dysfunction**				
Week 2		6 (11.1%)	8 (14.8%)	0.45
Week 26		6 (11.1%)	15 (28.8%)	0.02
**Chronic pelvic pain**				
Week 2		0	0	1.00
Week 26		0	1 (1.9%)	0.303

AC, abdominal circumference; EA, electroacupuncture; BMI, body mass index; SD, Standard Deviation; HC, hip circumference; IQR, Inter-Quartile Range; IRD= inter recti distance; SAT, subcutaneous adipose tissue; F%, percentage body fat; PF, physical functioning; RP, role-physical; BP, bodily pain; GH, general health; VT, vitality; SF, social functioning; RE, role-emotional; MH, mental health; HT, health transition.

* Data for 106 patients (54 randomized to the EA group and 52 to the control group) were used in the final analysis.

# Comparisons of means within group.

## Comparisons were carried out between groups.

† The mean value.

‡ The difference.

The pelvic girdle includes inguinal, pubic symphysis, coccyx, sacrum, and either side of the pelvis.

§ The midpoint of umbilicus and xiphoid process.

Δ At the supine position.

At 26 weeks follow-up, the mean of SAT in paraumbilical and right triceps of the two groups in both groups were reduced compared with those before treatment at 26 weeks, and the difference was statistically significant (*P* < 0.05). The SAT difference in paraumbilical was reduced in the EA group than in the control group on the front-to-back difference between groups, with statistical significance (*P* < 0.05) (Table 4). The comparison between groups suggested that picking pain was less frequent in the EA group than in the control group and the difference was statistically significant (*P* < 0.05). VAS scores for the difference between follow-up and post-treatment in the EA group, and present pain intensity (PPI) for the difference between follow-up and post-treatment in the control group all decreased and were statistically different (*P* < 0.05). The comparison between the two groups suggested that there was no statistical difference in the total EPDS score between the two groups (*P* > 0.05). Within-group comparisons suggested a statistically significant difference between the two groups after follow-up and before treatment (*P* < 0.05), but there was a statistically significant difference between the EA group after follow-up and after treatment (*P* < 0.05), and no statistically significant difference between the control group after follow-up and after treatment (*P* > 0.05) (Table 5).

At the end of treatment and the end of follow-up, within-group comparisons suggested a statistical difference between the two groups in terms of total MBIS and HerQles scores after treatment (*P* < 0.05), but between-group comparisons suggested no statistical difference between the two groups (*P* > 0.05) (Table 5).

In DRA-related symptom assessment, there was no statistical difference in any of the symptoms after treatment (*P* > 0.05) at week 2. The EA group was better than the control group in the improvement of sexual dysfunction at week 26, and the difference was statistically significant (*P* < 0.05); constipation, chronic pelvic pain, and urine leakage, frequency, and urgency, were not statistically significant (*P* > 0.05) at week 26 (Table 5).

In addition, exploratory subgroup and *post hoc* analyses were performed to determine whether cesarean delivery was performed. The study found that the EA group had a statistically significant difference in IRD at the midpoint of umbilicus and xiphoid process in head-up and flexed knee state, cm Δ† compared to the control group (OR = 0.904, 95% CI: 0.820-0.998, *P* = 0.046 < 0.05) (Supplementary material 5).

## Discussion

This randomized, controlled clinical trial was carried out at Hangzhou Hospital of Traditional Chinese Medicine, Hangzhou, China.

DRA is a common complication during pregnancy and the postpartum period. Postpartum DRA may cause a decrease in the tension of the elastic LA, resulting in a decrease in the ability to transmit abdominal forces across the midline, which in turn may affect abdominal muscle function. A severe decrease in the tension of the elasticity of LA may cause bulging of the abdominal organs, which may alter the appearance of the abdomen, seriously affecting the aesthetics of the body and reducing the woman's perception of her self-image. Postnatal DRA reduces the strength of the abdominal muscles and significantly reduces the support for the low back, affecting the mechanical balance of the low back muscles (7), resulting in a tilted pelvis, increasing the physiological curvature of the lumbar spine and increasing the incidence of low back pain and accumulation of abdominal fat. Therefore, postpartum DRA presents both psychological and physiological obstacles to the mother. However, there is a lack of ideal treatment options for postpartum DRA, and existing treatments such as physical rehabilitation, electrophysiological stimulation, and surgical treatment are still being developed. EA originating from traditional acupuncture around the 1930s has been verified to significantly improve the therapeutic effects of traditional acupuncture in a variety of diseases (31). This randomized trial showed that, compared with the control group, 10 sessions of EA for 2 weeks provided a higher improvement in IRD, especially at the horizontal line of the umbilicus in the end-expiratory state. Physical exercise has therapeutic effects on activation and induces transverse abdominis contraction and tightening of LA, and the different values of IRD are all changed, but the more obvious effect of the EA group may be related to EA therapy and the selection of acupoints.

The abdominal selection of acupoints treated in this study include bilateral Tianshu (ST25) and bilateral Dai Mai (GB26) at the horizontal line of the umbilicus, but Zhongwan (RN12) and Xiawan (RN10) in linea alba at the midpoint of umbilicus and xiphoid processor. Qi Hai (RN6), and Guanyuan (RN4) in linea alba at the midpoint between the umbilicus and pubic symphysis, may be related to the number of acupoints and curative effect (32). IRD was measured in the end-expiratory state to assess the width of the abdominal linea alba under transverse abdominis contraction.

The difference in IRD was only in the end-expiratory state, which might be related to the activation and enhancement of transverse abdominis tension by physical exercise and the EA group. The rectus sheath wraps the rectus abdominal muscles and is divided into two layers: the anterior is formed by the healing of the aponeurosis of the external oblique muscle and the aponeurosis of the internal oblique muscle, and the posterior rectus sheaths are formed by the healing of the aponeurosis of the internal oblique muscle and the aponeurosis of the transverse abdominis. The posterior rectal sheath is functionally more related to transverse abdominis than rectus abdominal muscles, and activation of transverse abdominis plays an important role in the etiology of the DRA (34, 35). Physical exercises adopted in this study, such as left and right-side leg rotation, are more effective in activating deep transverse abdominis, external oblique muscle, and internal oblique muscle (36, 37), and posterior rectus fascia sheath formed by transverse abdominis tendon sheath has better efficacy in maintaining abdominal wall tension stability. On the other hand, previous studies (38) found that electro-acupuncture had a more significant activation effect on transverse abdominis, which accelerated the adjustment of alba and transverse abdominis fascia tension to the normal level on the basis of rehabilitation exercise. However, it may not be reflected due to insufficient sample size or a short course of EA.

In addition, to further determine whether there are other influencing factors, such as fascia tension imbalance of transverse abdominis, fascia tension imbalance of muscles around the linea alba, or fascia tension imbalance of pelvic floor muscles, we added the normal population as a control group (Supplementary material 4) and found that these factors were present in the end-expiratory state compared with the normal population. These unbalance factors were corrected by EA; IRD and pelvic floor muscle status were improved. Therefore, the EA group was superior to the control group in improving IRD at all sites and states at 26 weeks. Only IRD at the midpoint of the umbilicus and xiphoid process in end-expiratory showed statistically significant changes, which to some extent indicated that EA corrected these imbalance factors and achieved long-term improvement.

Previous studies have shown that women with DRA in the first year postpartum have a significantly lower trunk muscle rotational moment and a significantly lower score on the sit-up test and that rectus abdominis spacing is negatively associated with trunk rotational moment and sit-up test scores (39). It is possible that these changes are related to the widening and thinning of the elasticity of LA during pregnancy, resulting in an imbalance in tension. When DRA occurs, the tension of the wide and thin LA decreases, the stabilization of the abdominal muscle and the conduction of abdominal wall force are reduced, and the abdominal wall is relaxed. Lee et al. (40) proposed the deformation index as a means of assessing the elasticity of LA, suggesting that the greater the deformation index the less elastic it is, whereas in this study the elasticity of LA was assessed by strain-based elastography. This study found that: In the control group, the elasticity of linea alba was smaller than that of the EA group at two sites (the horizontal line of the umbilicus, and the midpoint of the umbilicus and xiphoid process) at week 2 and week 26 (*P* < 0.05). In terms of the correlation between the elasticity of linea alba and IRD, the elasticity of the LA score was negatively correlated with IRD (rs = −0.356, *P* < 0.05) (Table 4). Beamish et al. (41) suggested that the elasticity of linea alba was worse when the IRD was greater in patients with DRA, which is consistent with the study. Reducing linea alba deformation and increasing the elasticity of linea alba or making this a goal is important for subsequent DRA rehabilitation.

The tension of the anterior abdominal wall (including the LA) in patients with DRA is influenced by the entire abdominal wall myofascia, and an imbalance in the tension of the anterior abdominal wall myofascia caused by DRA can also cause changes in the tension of the entire abdominal myofascia (42). The specific morphological alterations of the lateral abdominal muscle groups and other muscle and fascia tissues throughout the body in patients with DRA have not been reported in the literature. Some studies have analyzed the correlation between DRA and abdominal muscle dysfunction, and a study by Liaw et al. (43) found that abdominal muscle function showed a negative correlation with the mean IRD, which is consistent with the results of the present study. Narrowing IRD may lead to an increase in trunk flexion and rotation strength and endurance to some extent.

In addition, this study takes into account that postpartum DRA causes changes in overall trunk biomechanics, which is more conducive to understanding the pathophysiological changes of DRA and clarifying the coordination and unity of the abdomen and pelvic floor (40), and that treatment cannot address only a single muscle or symptom. Even the combined thoracoabdominal breathing of the control group is called to emphasize the opening of the thorax and the inward retraction of the abdomen. Combined with the elastography results, comparison between groups suggested an advantage of the elasticity of linea alba in the EA group compared to the control group (*P* < 0.05) at week 2, this could provide an important basis for the improvement of rectus abdominis spacing, i.e., the improvement of elasticity in the short term and an improvement of distance in the long term. The same is true for the pelvic floor results, both at 2 weeks and 26 weeks, the EA group showed an improvement compared to the control group, except that the results were more significant in slow muscle during systole (*P* < 0.05). The same is true for the electromyographic evaluation of pelvic floor results, where both at 2 and 26 weeks, the EA group showed an improvement compared to the control group, except that the results were more significant in the mean value of slow muscle during systole (*P* < 0.05). Postpartum women often have pelvic floor dysfunction, and there is no consensus on whether DRA is associated with pelvic floor dysfunctional disorders (44, 45). In the supine position with low intra-abdominal pressure, contraction of the abdominal musculature activates contraction of the pelvic floor musculature (46), as advocated in the control group with combined thoraco-abdominal breathing, emphasizing the opening of the thorax and the internal retraction of the abdomen. The “abdominal tank” theory suggests the coordination and unity of the abdomen and pelvic floor (40) and that treatment should not address only a single muscle or symptom. EA enhances pelvic floor innervation and muscle support (47), thereby improving pelvic floor muscle strength.

In general, obesity is determined by the body mass index (BMI) (48). BMI has been suggested as a possible risk factor for DRA, due to excess fat in the abdominal cavity exerting excessive pressure on the abdominal wall, thus causing further separation of DRA on both sides (49). And on the other hand, it has been suggested that muscle loss may co-exist (50), thus raising the idea that obese people are more likely to have DRA (51). The results of the study showed that the EA group was better than the control group at reducing BMI and when the patients' DRA treatment improved, BMI was also reduced compared to the previous one. The paraumbilical SAT and F% better represent the fat distribution of the body. DRA reduces the strength of the abdominal muscles and significantly reduces the support for the low back, affecting the mechanical balance of the low back muscles, increasing the accumulation of abdominal fat, and increasing the paraumbilical SAT (52). Therefore, postnatal DRA, in turn, increases the degree of abdominal laxity, affecting the aesthetics of the shape. The interconnection between the separation of the rectus abdominis muscle and abdominal obesity affects each other.

Postpartum abdominal skin laxity is a natural manifestation of skin aging and may be associated with increased skin collagen gaps, weak skin elastic fibers, and weak skin contraction (53). The maternal experience of pregnancy and childbirth causes mechanical strain on the abdominal muscles, especially the rectus abdominis, resulting in increased muscle tension and poor elasticity. EA can reduce the muscle tension of the abdominal muscles in patients with rectus abdominis detachment by using the corresponding points in the abdomen, increasing the proportion of type I collagen and a decrease in the proportion of type III collagen in the tendon fascia, thus causing a change in the expression form of collagen and achieving a repair of the damaged rectus abdominis muscle (54, 55). The EA helps to improve abdominal laxity by inhibiting the expression of pro-inflammatory cytokines (TGF-β1), allowing the TGF-β1/CTGF pathway to function properly and promoting the regeneration of myoelastin fibers (56).The probability of persistent abdominal laxity in the postpartum period is 30–40%. Pregnancy and childbirth cause the LA to widen and weaken, and the abdominal skin to loosen and sag and bulge in the midline, making the abdominal core unstable and leading to low back pain (1). During the SF-MPQ analysis, we found that pick pain was less frequent in the EA group than in the control group in terms of long-term effects (at week 26) and the difference was statistically significant (*P* < 0.05).

When DRA is studied, some scholars examine the interrelationship between diseases, and the abdominal canal theory (40) considers other symptoms of the abdominopelvic muscles when describing pelvic-abdominal coordination, thus linking the mechanisms of disease occurrence in tandem, or forming a hypothesis. Indigestion, low back pain, postpartum depression, quality of life, and menstrual changes are common problems in postpartum women, but the association with DRA is unknown (1, 51, 57), so this study continues to develop reported research on these factors. LDQ scores suggested that digestive symptoms were better in the EA group than in the control group both after treatment and after follow-up, and the improvement was more prominent in the symptoms of belching. Menstrual symptoms scores suggested that 24 weeks after the end of treatment, 38 people in the rehabilitation group and 39 people in the acupuncture group had menstruated. Comparison between groups suggested no significant difference in menstrual symptoms between the two groups. Due to the need to breastfeed during the puerperium, not all postpartum women's menstruation returned, so a pre- and post-group comparison was not possible. It has also been shown Gluppe et al. (58) that after 10 sessions of conventional Tui Na combined with physiotherapy for postpartum DRA, the patient's IRD shrank and QoL improved significantly, and no recurrence or worsening of postpartum DRA was found after more than 12 weeks of follow-up. The SF-36 score was for 1 month, and 1 month after the end of 10 treatments partially overlapped in time with the first filling, so we chose to compare during the follow-up period with the pre-treatment period, reflecting the fact that QoL in women with postpartum DRA was 24 weeks after the end of treatment compared to the pre-treatment period A trend toward improvement, especially PF was significantly improved. The degree of improvement in daily functional limitations treated with EA was better than in the control group, with better results for the long-term effects of EA. The health status of patients at 24 weeks after the end of treatment correlated with the presence of DRA at 24 weeks after the end of treatment.

Some researchers have investigated the correlation between DRA and low back pain, with Sperstad et al. (1) reporting no difference in the incidence of chronic lower back pain and pelvic girdle pain between DRA and non-DRA patients. EA could effectively activate the TrA, RA, and internal and external oblique abdominal muscles, promote the restoration of proprioception, release the fascia, and accelerate the improvement of muscle strength and elasticity repair of the abdominal muscles. The study effectively reflected whether the patients' current low back pain was caused by pain or by psychological effects using the SF-MPQ. The results showed an improvement in both groups compared to pre-treatment, with less pain provocation in the follow-up period after EA treatment than in the control group, indicating an advantage of EA in improving low back pain and a more pronounced long-term effect of EA treatment. The changing role of women in modern society requires them to recover quickly after childbirth and integrate into society, and the physical changes brought about by pregnancy often cause psychological changes. The impact of the physical changes brought about by DRA on maternal self-perception and emotions is of concern. EPDS and MBIS scores were significantly better in both groups after treatment and at follow-up, but the difference between the two was not significant, suggesting an improvement in postnatal depression and self-image valuing issues regardless of the method, although the efficacy outcomes were similar.

Patients with DRA have a wider and thinner LA, a reduced elastic component, and decreased tension, resulting in a reduction in the ability to transmit abdominal muscle forces across the midline (40), affecting abdominal wall morphology and abdominal muscle function. In this study, HerQles scores were found to be significantly better in both groups after treatment and at follow-up, but the difference between the two was not significant, suggesting that abdominal wall valuation problems improved regardless of the method used, although the efficacy results were similar. Postpartum-related symptoms (leakage, constipation, urinary frequency, urgency, sexual dysfunction, changes in chronic pelvic pain) were extracted from previous literature (1) and used to see if there was a correlation between DRA and the following symptoms. However, the results of the study suggested that no significant differences were seen between the two. This is consistent with the findings of previous scattered literature (1, 57, 58). In contrast, symptoms of sexual dysfunction were less frequent in the EA group after treatment than in rehabilitation during follow-up, suggesting that EA has a unique advantage in this regard.

The study aimed to determine whether EA was effective in DRA, we wanted to find out further during the study whether it would be more effective in patients who had a cesarean delivery, given that the presence or absence of a cesarean delivery might interact with the trial intervention (p for interaction), an exploratory subgroup analysis was conducted based on the presence or absence of cesarean delivery. It was found that there was an interaction between the presence or absence of cesarean delivery compared to the control group on the difference in umbilical level flexion at follow-up and that EA had a more significant improvement in IRD in patients who had a cesarean delivery, which may be more applicable to patients who had a cesarean delivery (*P* < 0.05).

This study investigated the therapeutic effect of EA combined with physical exercise on postnatal DRA and compared it with only physical exercise to objectively evaluate the clinical efficacy of both on postnatal DRA from multiple perspectives. The study provides an objective evaluation, guidance, and new ideas and methods for the clinical treatment of postpartum DRA, and will have scientific significance and practical value for the study of DRA and the promotion of EA.

## Limitation

1. Random errors during the trial: (1) Unavoidable individual differences, e.g., frequency intensity of EA parameters were selected between 4 and 6 because of individual tolerance differences. (2) Errors caused by uncontrollable factors in the research process, e.g., since the treatment was not blind to patients, we could not rule out that the clinical improvement in DRA was due to the expected value or placebo effect. In addition, although the therapist did not know the purpose of the experiment and did not have knowledge of acupuncture and moxibustion, she performed the blind method. However, some marks were left on the abdomen after electro-acupuncture, so the patient received rehabilitation treatment first and then electro-acupuncture. 2. Selection bias: Berkson rate bias due to the single-center study. 3. Recall bias of patients with DRA: as the questions in the questionnaire involved the collection of past information, the research results were biased due to the incomplete memory of the subjects. 4. Confounding bias may exist during subgroup analysis, because subgroup analysis of trials neutralizes the benefits of randomization, which leads to potentially biased results (59). 5. Because of the degree of bladder filling, the patient's position has been shown to affect the results of the measurements. In addition, it is occasionally difficult to obtain a valid Valsalva maneuver, so there is no clear and uniform reference measurement to date. 6. Examination means. Although ultrasound is a cost-effective and confirmatory means of detecting IRD, the results are influenced by the ultrasonographer's experience and the angle of incision of the ultrasound placement, the measurement duration, and despite the availability of intercepted images as evidence, it is not possible to observe the respiratory coordination. The muscle changes during the movement were not observed, and a way of monitoring dynamic changes was lacking.

## Conclusion

Compared with the control group (only physical exercise), ten sessions in the EA group for 2 weeks resulted in improvement in IRD, electromyographic evaluation of the pelvic floor, WHR, the elasticity of LA, paraumbilical SAT, symptoms of DRA, abdominal tension, and strengthening of abdominal muscles with durable effects 26 weeks.

## Data availability statement

The original contributions presented in the study are included in the article/Supplementary material, further inquiries can be directed to the corresponding author.

## Ethics statement

The studies involving human participants were reviewed and approved by the Ethics Committee of Hangzhou Hospital of Traditional Chinese Medicine reviewed this study protocol and gave its approval and consent (Approval Code 2020KY082). The patients/participants provided their written informed consent to participate in this study.

## Author contributions

YL: data analyses, figure preparation, and manuscript preparation. YZ: recruit subjects. LYJ: responsible for the design of randomization, project funding, and study initiation. CL and MYG: ultrasound evaluation. LJX: responsible for the design of randomization. LS: random allocation. JYC and TW: responsible for manual data measurement before treatment. LJD: physical therapists. YYS: acupuncture treatment. TTZ and MF: responsible for guidance and statistics. All authors approved the final version of the manuscript.

## Funding

This RCT is funded by the construction fund of medical key disciplines of Hangzhou (Project Number: OO20200097), Hangzhou medical and health science and technology project No. A20200483, and Zhejiang Traditional Chinese Medicine Science and Technology Plan Project (Project Number: 2021ZQ065).

## Conflict of interest

The authors declare that the research was conducted in the absence of any commercial or financial relationships that could be construed as a potential conflict of interest.

## Publisher's note

All claims expressed in this article are solely those of the authors and do not necessarily represent those of their affiliated organizations, or those of the publisher, the editors and the reviewers. Any product that may be evaluated in this article, or claim that may be made by its manufacturer, is not guaranteed or endorsed by the publisher.

## Abbreviations

AE: Adverse Event
BMI: Body Mass Index
DRA: Diastasis Recti Abdominis
OR: Odds Ratio
PASS: Power Analysis and Sample Size
IRD: Inter Recti Distance
PRI: Pain Rating Index
PPI: Present Pain Intensity
WHR: Waist-to-Hip Ratio
LDQ: Leeds Dyspepsia Questionnaire
MDQ: Menstrual Distress Questionnaire
SF-36: The MOS Item Short Form Health Survey
PT: physical therapists
LA: Linea Alba
SF-MPQ-2: Short-Form McGill Pain Questionnaire-2
EPDS-10: 10 Items of Edinburgh Postnatal Depression Scale
MBIS: The Modified Body Self-Image Scale
ICIQ-SF: International Consultation Incontinence Questionnaire Short-Form
HerQles: Hernia-Related Quality-of-Life Survey
SE: Side Effects.
